# Bridging the gap: development of a methodology for retrieving and harmonising body mass index (BMI) from population-level linked electronic health records

**DOI:** 10.1136/bmjopen-2025-103724

**Published:** 2025-10-05

**Authors:** Michael Jeanne Childs, Sarah J Aldridge, Helen Daniels, Gareth Ivor Davies, Victoria Best, Hoda Abbasizanjani, Ronan Lyons, Ashley Akbari, Fatemeh Torabi

**Affiliations:** 1 Population Data Science, Swansea University Medical School, Swansea, UK; 2 Dementias Platform UK, Swansea, UK; 3 Professional and Continuing Education, University of Cambridge, Cambridge, UK

**Keywords:** Body Mass Index, Electronic Health Records, PUBLIC HEALTH, EPIDEMIOLOGY

## Abstract

**Abstract:**

**Objective:**

This study aims to develop a methodology to retrieve, harmonise and evaluate the completeness of national body mass index (BMI) data from linked electronic health record (EHR) sources to build a longitudinal research-ready data asset (RRDA).

**Design:**

A longitudinal study of BMI records spanning 23 years (1 January 2000 to 31 December 2022) from four data sources.

**Setting:**

The national BMI RRDA is created within the Secure Anonymised Information Linkage (Databank), encompassing the entire population of Wales, UK.

**Procedure and participants:**

We built a methodology that provides a reproducible framework for extracting and harmonising BMI data from four major linked EHRs across two age groups: children and young people (CYP; 2–18 years old) and adults (19 years and older). The methodology is adaptable across different trusted research environments. We evaluated the completeness and retention of records over 1-, 5- and 23-year periods by calculating the proportion of missing data relative to each year’s population.

**Results:**

We retrieved 53.4 million records for 3.2 million individuals across Wales from 1st January 2000 to 31 December 2022. Among these, 3% of CYP and 34% of adults had repeat BMI measurements recorded over periods ranging from 5 to 23 years. Throughout the entire population of Wales during this period, 49% of CYP and 26% of adults had at least one BMI reading recorded, resulting in a missingness rate of 51% for CYP and 74% for adults. Preserving BMI information by retaining the most recently recorded BMI over 1-, 5- and 23-year intervals from 2022 showed coverage rates of 10%, 33% and 68%, respectively, for CYP, and 25%, 51% and 73%, respectively, for adults.

**Conclusions:**

Our findings highlight substantial variations in BMI data availability and retention across CYP and adults, as well as time periods within EHR in Wales. Wider adoption of this approach can enhance standardised approaches in using accessible measures like BMI to assess disease risk in population-based studies, strengthening public health initiatives and research efforts.

**Strengths and limitations of this study**

- Incorporated additional data sources for the retrieval of body mass index (BMI) for a population.
- Created a reproducible methodology for reliable retrieval and allocation of BMI categories.
- Increased the proportion of known BMI using historical data from electronic health records.
- Data used in this study is primarily for the Welsh population; however, we provide a workflow that could be reproduced in other trusted research environments.
- Limited by data available in the sources, reflecting the need to increase routine collection of BMI data in the population.

## Introduction

 According to the WHO, each year, there are approximately 3 million global fatalities attributed to overweight or obesity.^1^ Body mass index (BMI) provides a primary metric, derived from height and weight calculations, that is widely used due to its simplicity, speed and cost-effectiveness in assessing individuals’ body size, while complementary approaches have also been suggested for measurements contributing to premature health.^2^

Providing access to accurate BMI measures at the population level facilitates comprehensive analysis and comparison of data trends, which is crucial for understanding population health dynamics and guiding targeted interventions aimed at alleviating the impact of obesity-related diseases both in adults and children^3^ as well as assessment of an individual’s long-term risk of cardiovascular disease,^4^ diabetes^5^ and cancer.^6^

BMI is routinely documented in the UK healthcare system; however, a notable proportion of missing data compromises the usability of these measurements for research conducted using electronic health records (EHRs). Achieving greater completeness and consistency in EHR BMI measurements remains an ongoing challenge.^7^ Moreover, BMI measurements in EHR are often recorded selectively, often during clinical encounters where weight is considered relevant, such as in cases involving comorbid conditions or those attending primary care for weight-related concerns.^5 8^ This results in BMI data being more complete for individuals with higher healthcare utilisation or specific clinical needs, introducing potential bias when using EHR-derived BMI data for population-level research.^9^,^11^ Consequently, reporting BMI categories across age groups should be approached with caution, as these data may overrepresent individuals who are older, sicker or already identified as being at risk. In England, approximately one-third of patients have their weight recorded each year, with an average interval of 2 years between repeat measurements.^8^ Those with repeated BMI readings were likely to be female, from lower socioeconomic backgrounds and with chronic health conditions,^8 12^ potentially skewing the representation of individuals in research studies.^13^

Strategies to improve BMI data coverage and completeness include preserving BMI records longitudinally for up to 5 or 10 years.^9 12^ Furthermore, a notable limitation in using BMI for health research is relying on a single source for BMI record retrieval.^10 14^ In Wales, the Secure Anonymised Information Linkage (SAIL) Databank holds information on population-wide health records, including data sources that contain different components (height and weight) of BMI. Using data linkage, we can collate these components and evaluate the completeness of BMI records when collated longitudinally from primary care, secondary care and other data sources.

Our study aims were threefold: first, we aimed to develop a methodology for retrieving and harmonising BMI from population-level linked EHR that researchers could readily use, allowing for reproducibility across different trusted research environments (TREs) and create a research-ready data asset (RRDA). Second, we wanted to determine whether incorporating other data sources and preserving BMI records for longer periods would affect the evident missingness level in EHR data. Third, we also quantified the impact of service interruption on record capturing by comparing the counts of BMI recording pre- and post-COVID-19.

## Methods

### Study design

This is an observational study where we developed a methodology that extracted BMI components from four data sources including height, weight, BMI values and BMI categories from primary care records from the Welsh Longitudinal General Practice dataset,^15^ diagnosis of obesity from hospital/secondary care records in the Patient Episode Dataset for Wales,^16^ height and weight measurements during pregnancy recorded in Maternity Indicators Dataset (MIDS),^17^ and height and weight from the child Measurement Programme in Wales recorded within National Community Child Health (NCCH) data. By using different data sources, we aim to increase the population coverage of BMI data for health research in Wales. All BMI records were linked and compiled into a general dataset that was attached to a population spine, producing a longitudinal national RRDA for BMI records of the entire Welsh population between 2000 and 2022. Thus, researchers could either run the methodology in their own cohort or use the RRDA to extract data, thereby increasing the reliability of the BMI data component across various health research studies. The codes used to implement this methodology can be found online (https://github.com/SwanseaUniversityDataScience/RRDA-BMI).

### Technical implementation

For the methodology, essential parameters that must be provided include: (a) study start and end dates, (b) data sources, (c) defined acceptable thresholds for records taken on the same day and change in BMI over time, (d) defined acceptable difference between dates of height and weight records (for children’s data) and (e) Read Version 2 and International Classification of Diseases, 10th revision (ICD-10) codes pertaining to BMI components (online supplemental material tables 1 and 2).

The methodology incorporated several stages of data cleaning. First, it removed BMI value outliers and duplicate records at the point of extraction, followed by standardising height and weight measurements to calculate BMI values from height and weight using kg/m^2^.^18^ Finally, it identifies inconsistencies in daily and overtime records.

**Figure 1:**
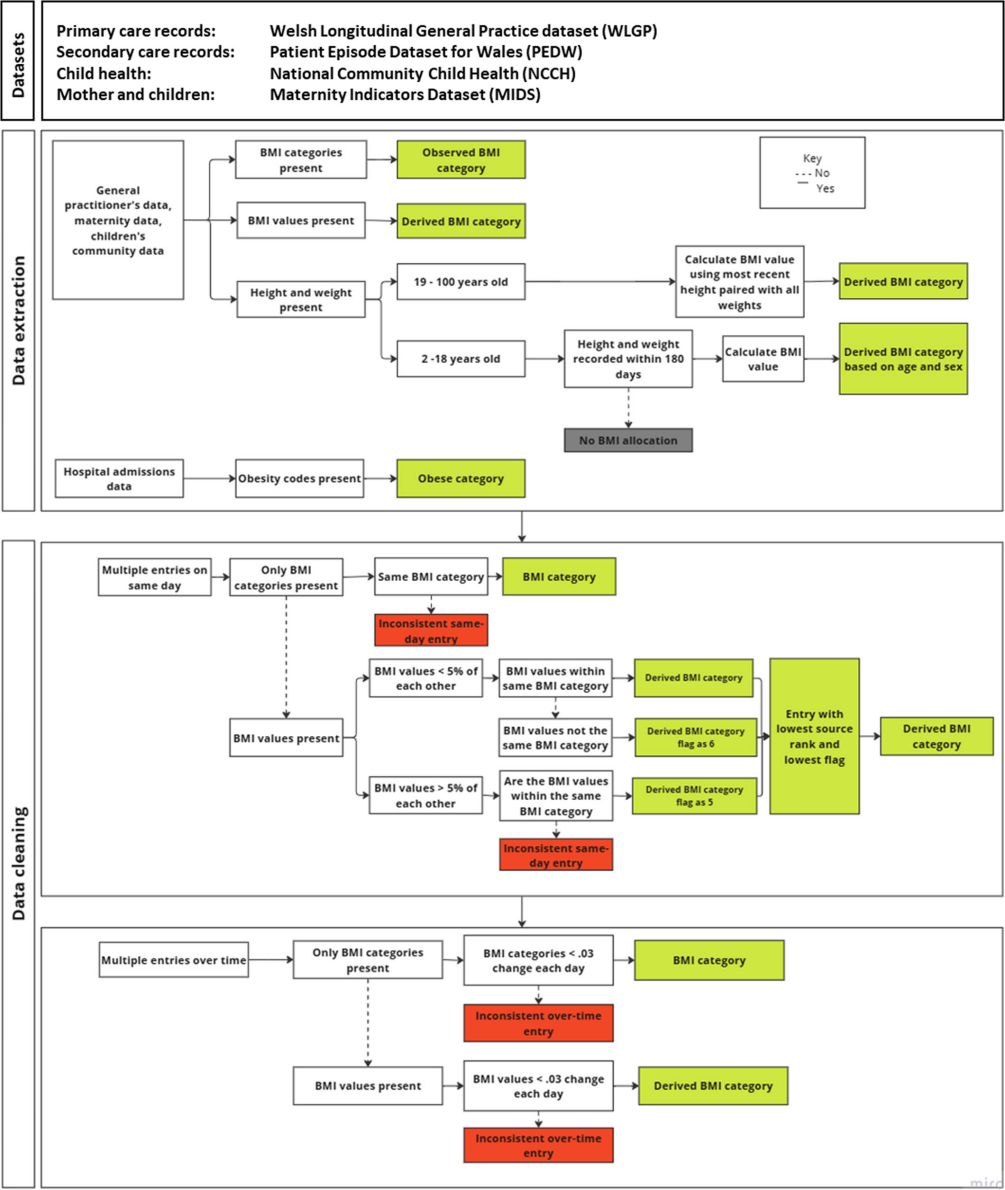
Implementation stages of methodology for the extraction and harmonisation of BMI records. Entries in green were kept, and entries in red in the data cleaning phase were removed. BMI, body mass index.

The records were compiled in a table of individuals with harmonised children and young people (CYP; 2–18 years old) and adult BMI categories (figure 1 and online supplemental material figure 1), which could be linked to a cohort of interest.

### Record retrieval from data sources

All data sources were longitudinal population-level data sources, spanning the entire study period (with MIDS from 2014 onwards), held within the national TRE for Wales, the SAIL Databank. All four data sources were used to retrieve BMI records for individuals residing in Wales, UK. We extracted all BMI components (eg, BMI categories, BMI values, height and weight, using READ codes (online supplemental material table 1) and diagnoses of obesity using ICD-10 codes (online supplemental material table 2), and compiled them into a reproducible and maintainable longitudinal RRDA containing harmonised BMI categories.

We also extracted individuals’ age, sex, ethnic group, week of birth (WOB), area of residence, start and end dates of residence, and date of death (DOD) where applicable from a combination of health, administrative^19 20^ and a linked harmonised ethnic group dataset compiled from primary care, secondary care and other data sources.^21^ To link the sources, we used a unique identifier known as the Anonymised Linkage Field (ALF), provided as part of a standard split-file anonymisation and linkage process.^22 23^ To ensure adequate linkage quality, we filtered for individuals with reliable deterministic matching only (ALF_STS_CD=1, 4, 39). For more information, see Lyons *et al*.^24^

### Cohorts

Individuals who were alive and living in Wales for more than 31 days from 1 January 2000 to 31st December 2022 were included in our cohorts: (a) *CYP*—BMI data recorded when individuals were aged 2–18 years old (inclusive, 24–228 months old), following WHO guidelines for BMI allocation purposes^25^ and (b) *adults*—BMI data recorded for individuals aged 19–100.

### Procedure

We extracted BMI-related data over 23 years for the population with general practitioners’ coverage in SAIL Databank^23 24^ using the following stages (see online supplemental material figure 1).

### Stage 1: data extraction and collation

The methodology extracted individual-level population-scale anonymised data detailing BMI components from 1 January 2000 to 31 December 2022 from the four health data sources mentioned separately and combined into a single longitudinal table, removing extreme BMI values (below 12 and over 100), height and weight. Multiple same-day entries from different data sources were permitted, and we used a combination of factors to rank the data types present (table 1).

**Table 1:** Hierarchy of BMI components applied to source types and data sources to prioritise the most relevant data sources for data available on a single day

Data source	Data type	Ranking	Rationale
WLGP	BMI value	1	Regular monitoring of BMI occurs at the primary care via direct input of BMI values in patient records^32^
WLGP	Height and weight	2	If direct BMI values were not found in the records, BMI has been calculated from recorded and cleaned heights and weights
MIDS	Height and weight	3	As above, but as a source of maternal data
NCCH	Height and weight	4	As above, but of children’s data
WLGP	BMI category	5	Primary care BMI category is used where BMI values were not recorded and not accessible through height and weight measurements
PEDW	Obese category	6	Secondary care data only captures the diagnosis of obesity and is used where this is a unique contribution to obese BMI categories

BMI, body mass index; MIDS, Maternity Indicators Dataset; NCCH, National Community Child Health; PEDW, Patient Episode Database for Wales; WLGP, Welsh Longitudinal General Practice.

### Stage 2: cohort construction and attaching demographic information

The methodology attached sex, WOB, DOD and residency period information for individuals with at least one BMI in stage 1. Those with no WOB record, invalid sex code, with DOD before the start of the study period and with DOD after 31 days of the study start date were removed. The DOD or date of moving out of Wales was a censoring point for adults, while for children, the date of their 19th birthday was considered as an end date for their contribution to the CYP branch of the methodology (online supplemental material figure 1).

### Stage 3: calculating age and BMI values

For CYP, height recordings for each individual were paired with all weights. To account for the rapid growth rate of children, the acceptable difference between height and weight record dates was limited to a default value of 180 days. BMI categories were assigned to the BMI values using percentiles and z-scores for the individual’s sex and age group at the BMI date. Due to several factors that may affect development during infancy, the use of BMI categories for children under 2 years old is not routine^26^ and hence not included.

For adults, we took the most recent height recorded and paired this with all the weights recorded in the study period. Calculated BMI values were then allocated a BMI category: underweight (<18.5), normal weight (18.5–24.9), overweight (25–29.9) and obese (>30).

### Stage 4: identifying inconsistent entries

In stage 4, when multiple records from different sources existed, we retained the BMI value from the source with the highest reliability, based on the source hierarchy detailed in table 1. A ‘lower rank’ refers to a more trusted or prioritised source in that hierarchy. All data were combined into one table with harmonised BMI categories. Duplicate BMI records for an individual were permitted to account for multiple sources. BMI categories were allocated a numerical value (underweight=1, normal weight=2, overweight=3 and obese=4) to allow for the identification of BMI category change within and between visits. For individuals with multiple BMI category recordings on the same day, values were ordered by ascending BMI and BMI category, that is, each measurement is listed in increasing order of BMI to help identify duplicates or inconsistencies.

We applied a two-stage cleaning process. We first cleaned for inconsistent same-day entries to address entry duplication. All entries were arranged in ascending order. For only those with BMI categories present, when BMI categories were different by one BMI category, these were both recorded as inconsistent and removed. For those with BMI values, entries with less than 5% difference and the same BMI categories were considered consistent and were kept. Next, we cleaned for inconsistent over-time readings, that is, BMI measurements recorded on different dates, to address longitudinal inconsistencies. For more details, see online supplemental material text 1.

### Stage 5: selection of a single entry per day and incorporation of pregnancy flags

The methodology removed inconsistent entries from stage 4 and kept the BMI data with the lowest hierarchy of source rank. Finally, we assigned a pregnancy flag indicating whether the BMI recorded was taken (±294 days, ie, 42 weeks) before or after a baby’s birth, using data from MIDS to highlight weight changes likely related to pregnancy. The result is a long table containing all BMI information, including data source and rank, with harmonised BMI category for each individual condensed to a single entry per date.

### Implementation of the BMI methodology in health data research context

To determine how many individuals have BMI records for the length of time they were in the study, we calculated each individual’s record contribution, starting at the study index date or the earliest available record in Wales. Missing residency dates were set to the start and end of the study. The length of data contribution to the study was calculated using residency information or DOD, whichever applied first, allowing us to get a proportion of individuals with BMI records relative to their study contribution length.

To determine the contribution of each data source and type to overall BMI data coverage, we looked at the counts of individuals with records from single data sources separately, and those with entries from more than one data source. To identify trends in BMI recordings and categories in adults and CYP across Wales between 2000 and 2022, we counted the number of individuals with a minimum of one record per year. When multiple BMI records were available for an individual in a particular year, we kept their most recent record.

Finally, to assess how assumptions about data availability over time affect BMI coverage estimates, we compared three distinct observation windows anchored to the end of the study (31 December 2022): preserving only the most recent entry from the past year (start date 1 January 2022), the past 5 years (start date 1 January 2018) and the full study period (start date 1 January 2000). This approach does not assume that BMI is routinely or universally recorded across the population; instead, it reflects how BMI data are often used in research.

### Patient and public involvement

This project is undertaken under a proposal submitted to the independent Information Governance Review Panel (IGRP), including members of the public (IGRP Project: 0911). Two public members contributed to the project’s approval as part of the IGRP panel.

## Results

For the 3.2 million current population of Wales,^27^ we constructed a longitudinal cohort representing 5.1 million unique individuals (1 717 111 CYP and 4 283 110 adults; due to the longitudinal nature, young people may contribute to both cohorts depending on their 19th birthday) who lived at any point in Wales between 2000 and 2022, contributing to a follow-up over a period of 23 years with average follow-up per person of 7 years.

The majority of CYP were white, aged 5–13 years old and from urban cities and towns. The proportion of missing BMI in 2022 was 90% (table 2). Similarly, the majority of adults were White, between 19–69 years old and from urban city and town areas. The proportion of missing BMI in 2022 was 76% (table 3).

**Table 2:** Characteristics of children and young people grouped by 5-year periods

Characteristic	2000–2004 n=872 176*	2005–2009 n=1 693 198*	2010–2014 n=1 749 609*	2015–2019 n=1 657 702*	2020–2022 n=1 219 806*
Sex					
Female	428 638 (49%)	848 740 (50%)	875 428 (50%)	820 341 (49%)	605 329 (50%)
Male	443 538 (51%)	844 458 (50%)	874 181 (50%)	837 361 (51%)	614 477 (50%)
Ethnic group					
Asian	16 101 (1.8%)	46 205 (2.7%)	57 281 (3.3%)	62 690 (3.8%)	48 319 (4.0%)
Black	3829 (0.4%)	14 531 (0.9%)	19 247 (1.1%)	21 121 (1.3%)	15 737 (1.3%)
Mixed	9827 (1.1%)	27 983 (1.7%)	37 403 (2.1%)	45 510 (2.7%)	36 332 (3.0%)
Other	3462 (0.4%)	13 394 (0.8%)	20 657 (1.2%)	26 299 (1.6%)	18 743 (1.5%)
Unknown	77 541 (8.9%)	35 777 (2.1%)	20 612 (1.2%)	24 972 (1.5%)	53 818 (4.4%)
White	761 416 (87%)	1 555 308 (92%)	1 594 409 (91%)	1 477 110 (89%)	1 046 857 (86%)
Age band					
2–5	127 093 (15%)	376 735 (22%)	407 038 (23%)	356 461 (22%)	259 212 (21%)
5–13	315 190 (36%)	652 006 (39%)	685 383 (39%)	685 764 (41%)	544 217 (45%)
13–19	429 893 (49%)	664 457 (39%)	657 188 (38%)	615 477 (37%)	416 377 (34%)
BMI category					
Underweight	6306 (0.7%)	12 612 (0.7%)	13 251 (0.8%)	16 248 (1.0%)	6317 (0.5%)
Normal weight	206 377 (24%)	381 341 (23%)	407 383 (23%)	464 281 (28%)	145 448 (12%)
Obese	24 682 (2.8%)	54 824 (3.2%)	55 782 (3.2%)	62 872 (3.8%)	24 217 (2.0%)
Overweight	35 689 (4.1%)	65 623 (3.9%)	64 393 (3.7%)	70 375 (4.2%)	22 540 (1.8%)
Unknown	599 122 (69%)	1 178 798 (70%)	1 208 800 (69%)	1 043 926 (63%)	1 021 284 (84%)
Welsh Index of Multiple Deprivation 2019					
Most deprived	194 494 (22%)	404 771 (24%)	424 378 (24%)	406 403 (25%)	297 316 (24%)
2	172 141 (20%)	343 546 (20%)	360 465 (21%)	336 684 (20%)	247 877 (20%)
3	170 831 (20%)	325 311 (19%)	334 131 (19%)	316 720 (19%)	232 244 (19%)
4	162 861 (19%)	308 785 (18%)	312 886 (18%)	297 726 (18%)	220 938 (18%)
Least deprived	171 849 (20%)	310 785 (18%)	317 749 (18%)	300 169 (18%)	221 431 (18%)
Rural-urban classification					
Rural town and fringe	115 533 (13%)	218 358 (13%)	222 448 (13%)	211 108 (13%)	156 475 (13%)
Rural town and fringe in a sparse setting	33 724 (3.9%)	64 319 (3.8%)	63 114 (3.6%)	57 239 (3.5%)	40 769 (3.3%)
Rural village and dispersed	56 170 (6.4%)	100 414 (5.9%)	100 117 (5.7%)	93 305 (5.6%)	69 061 (5.7%)
Rural village and dispersed in a sparse setting	61 798 (7.1%)	111 697 (6.6%)	111 346 (6.4%)	104 010 (6.3%)	74 699 (6.1%)
Urban city and town	584 825 (67%)	1 161 266 (69%)	1 212 866 (69%)	1 155 601 (70%)	853 630 (70%)
Urban city and town in a sparse setting	20 126 (2.3%)	37 144 (2.2%)	39 718 (2.3%)	36 439 (2.2%)	25 172 (2.1%)

* n (%).

BMI, body mass index.

**Table 3:** Characteristics of adult population grouped by 5-year periods

Characteristic	2000–2004 n=2 725 063*	2005–2009 n=2 827 997*	2010–2014 n=2 912 460*	2015–2019 n=2 974 773*	2020–2022 n=2 860 214*
Sex					
Female	1 392 657 (51%)	1 434 743 (51%)	1 465 917 (50%)	1 497 773 (50%)	1 440 131 (50%)
Male	1 332 406 (49%)	1 393 254 (49%)	1 446 543 (50%)	1 477 000 (50%)	1 420 083 (50%)
Ethnic group					
Asian	29 617 (1.1%)	55 409 (2.0%)	78 360 (2.7%)	86 071 (2.9%)	85 070 (3.0%)
Black	7176 (0.3%)	13 777 (0.5%)	19 036 (0.7%)	22 491 (0.8%)	23 135 (0.8%)
Mixed	8823 (0.3%)	14 106 (0.5%)	19 405 (0.7%)	23 817 (0.8%)	24 440 (0.9%)
Other	6855 (0.3%)	14 631 (0.5%)	23 479 (0.8%)	29 945 (1.0%)	29 614 (1.0%)
Unknown	587 584 (22%)	365 161 (13%)	196 950 (6.8%)	172 191 (5.8%)	174 506 (6.1%)
White	2 085 008 (77%)	2 364 913 (84%)	2 575 230 (88%)	2 640 258 (89%)	2 523 449 (88%)
Age band					
19–29	515 218 (19%)	559 762 (20%)	574 367 (20%)	552 264 (19%)	493 500 (17%)
30–39	481 736 (18%)	445 764 (16%)	445 875 (15%)	474 500 (16%)	466 468 (16%)
40–49	454 789 (17%)	488 042 (17%)	468 434 (16%)	426 194 (14%)	409 758 (14%)
50–59	424 667 (16%)	418 316 (15%)	452 304 (16%)	484 537 (16%)	474 985 (17%)
60–69	338 365 (12%)	394 089 (14%)	414 408 (14%)	410 600 (14%)	420 239 (15%)
70–79	268 353 (9.8%)	272 787 (9.6%)	298 659 (10%)	350 834 (12%)	351 666 (12%)
80–89	181 104 (6.6%)	187 032 (6.6%)	184 135 (6.3%)	196 531 (6.6%)	185 583 (6.5%)
90 and over	60 831 (2.2%)	62 205 (2.2%)	74 278 (2.6%)	79 313 (2.7%)	58 015 (2.0%)
BMI category					
Underweight	27 662 (1.0%)	37 807 (1.3%)	41 955 (1.4%)	44 640 (1.5%)	29 560 (1.0%)
Normal weight	415 164 (15%)	503 287 (18%)	494 920 (17%)	468 033 (16%)	296 591 (10%)
Obese	291 760 (11%)	436 690 (15%)	485 704 (17%)	535 455 (18%)	423 655 (15%)
Overweight	392 586 (14%)	506 440 (18%)	506 457 (17%)	497 688 (17%)	338 334 (12%)
Unknown	1 597 891 (59%)	1 343 773 (48%)	1 383 424 (48%)	1 428 957 (48%)	1 772 074 (62%)
Welsh Index of Multiple Deprivation 2019					
Most deprived	515 681 (19%)	534 122 (19%)	547 889 (19%)	563 271 (19%)	546 372 (19%)
2	531 214 (19%)	550 926 (19%)	564 975 (19%)	572 171 (19%)	552 224 (19%)
3	578 052 (21%)	601 338 (21%)	622 489 (21%)	636 234 (21%)	603 979 (21%)
4	549 697 (20%)	572 127 (20%)	587 802 (20%)	603 738 (20%)	582 577 (20%)
Least deprived	550 419 (20%)	569 484 (20%)	589 305 (20%)	599 359 (20%)	575 062 (20%)
Rural-urban classification					
Rural town and fringe	351 191 (13%)	361 773 (13%)	369 702 (13%)	375 655 (13%)	366 228 (13%)
Rural town and fringe in a sparse setting	113 863 (4.2%)	116 259 (4.1%)	117 270 (4.0%)	118 189 (4.0%)	111 419 (3.9%)
Rural village and dispersed	186 000 (6.8%)	191 868 (6.8%)	194 537 (6.7%)	198 230 (6.7%)	193 214 (6.8%)
Rural village and dispersed in a sparse setting	210 883 (7.7%)	218 808 (7.7%)	223 732 (7.7%)	227 516 (7.6%)	219 995 (7.7%)
Urban city and town	1 800 831 (66%)	1 875 012 (66%)	1 941 431 (67%)	1 991 566 (67%)	1 912 809 (67%)
Urban city and town in a sparse setting	62 295 (2.3%)	64 277 (2.3%)	65 788 (2.3%)	63 617 (2.1%)	56 549 (2.0%)

* n (%).

BMI, body mass index.

**Figure 2:**
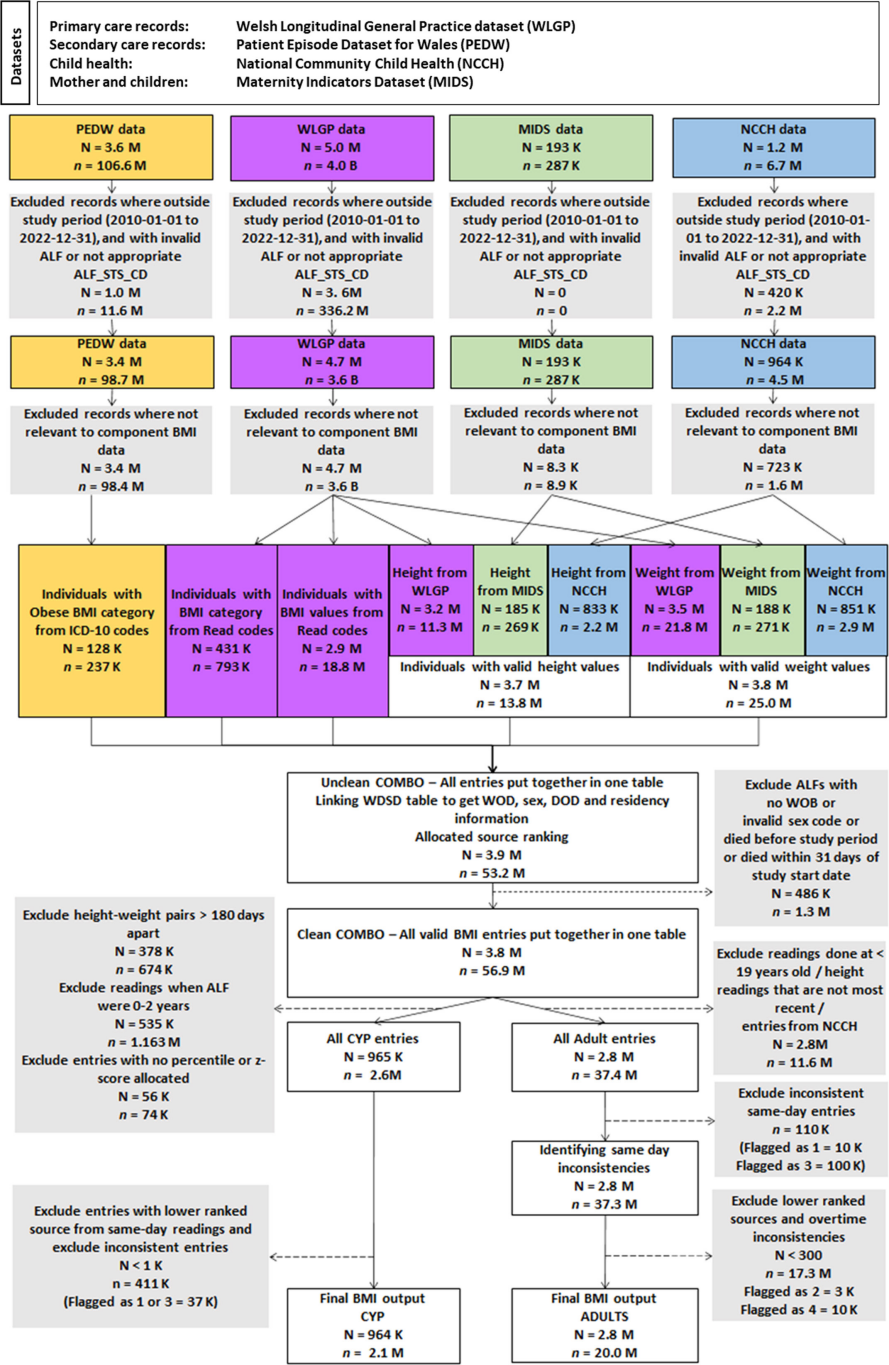
Selection and exclusion process used in the methodology. ALF, Anonymised Linkage Field; BMI, body mass index; CYP, children and young people; DOD, date of death; ICD-10, International Classification of Diseases, 10th revision; N, unique individuals, n, total entries; WDSD, Welsh Demographic Service Dataset; WOB, week of birth; WOD, week of death.

**Figure 3:**
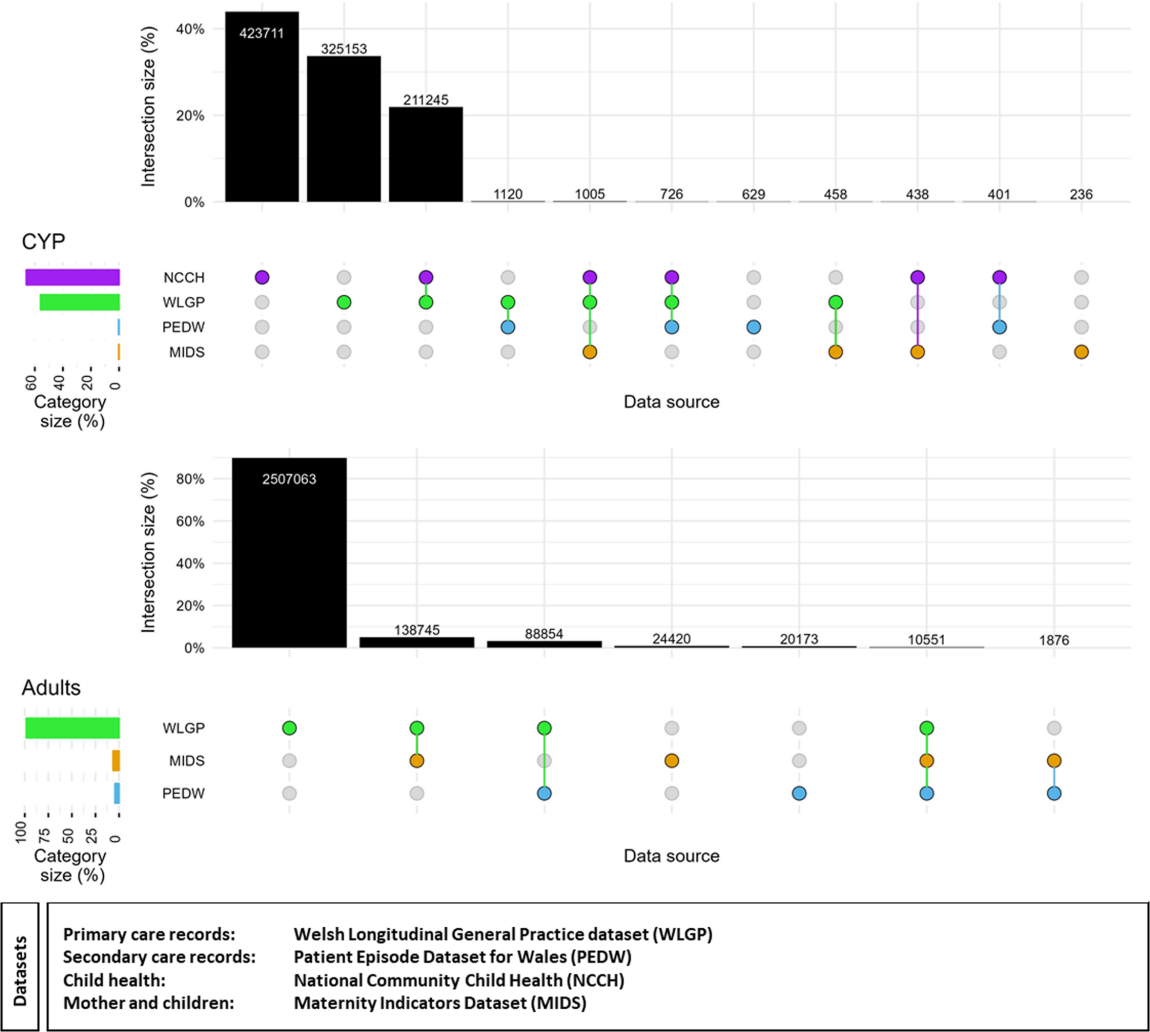
Distribution of data sources across the study population and percentage overlap among individuals with records in multiple sources. An upset plot has three components. The bar chart at the top shows the count of the population which have data from a particular combination of sources, each bar representing a different type of combination. The graphical bar underneath represents the combinations, where each row is a data source, where the dots and lines show the combinations of data sources for each subset. Finally, the smaller bar plot on the left of the graphical table shows the unconditional count of individuals from each data source. CYP, children and young people.

Using phenotyped BMI categories, as recorded in patients’ primary care dataset using READ codes, we identified 431 156 unique individuals (8.4% of the total population) between 2000 and 2022. Using the methodology, a total of 2.1 million entries were extracted and curated for 964 328 (56.2%) CYP and 20.0 million entries for 2 791 404 (65.2%) adults. Figure 2 shows the selection and exclusion process used in the methodology and the counts of individuals with BMI information. The methodology pulls together all contributing factors into BMI from existing primary care, secondary care, maternity and children’s community data sources. Children’s community health data and primary care sources contributed the most to the overall BMI data (figure 3). We observed an increase of 44% in unique individuals with calculated BMI values from children’s community health data and .1% from hospital admissions and maternity data combined across the whole study period. For the adult cohort, 98% of the overall BMI data were from primary care, and <2% from hospital admissions and maternity data.

**Figure 4:**
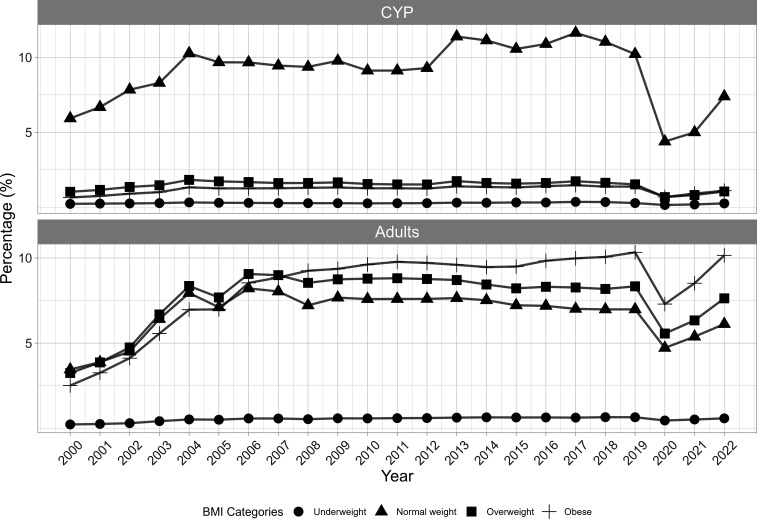
Frequency of known BMI categories in Wales between 2000 and 2022 in CYP and adults. The most recent category for the year is presented. BMI, body mass index; CYP, children and young people.

Of the known yearly BMI categories from 2000 to 2022 (figure 4), the majority of the CYP were categorised as normal weight (804 153, 47%), followed by overweight (180 188, 10%), obese (120 783, 7%) and underweight (37 343, 2%). For adults, the majority were overweight and normal weight (both 1.4 million, 33%), obese (1.1 million, 27%) and underweight (173 954, 4%).

**Figure 5:**
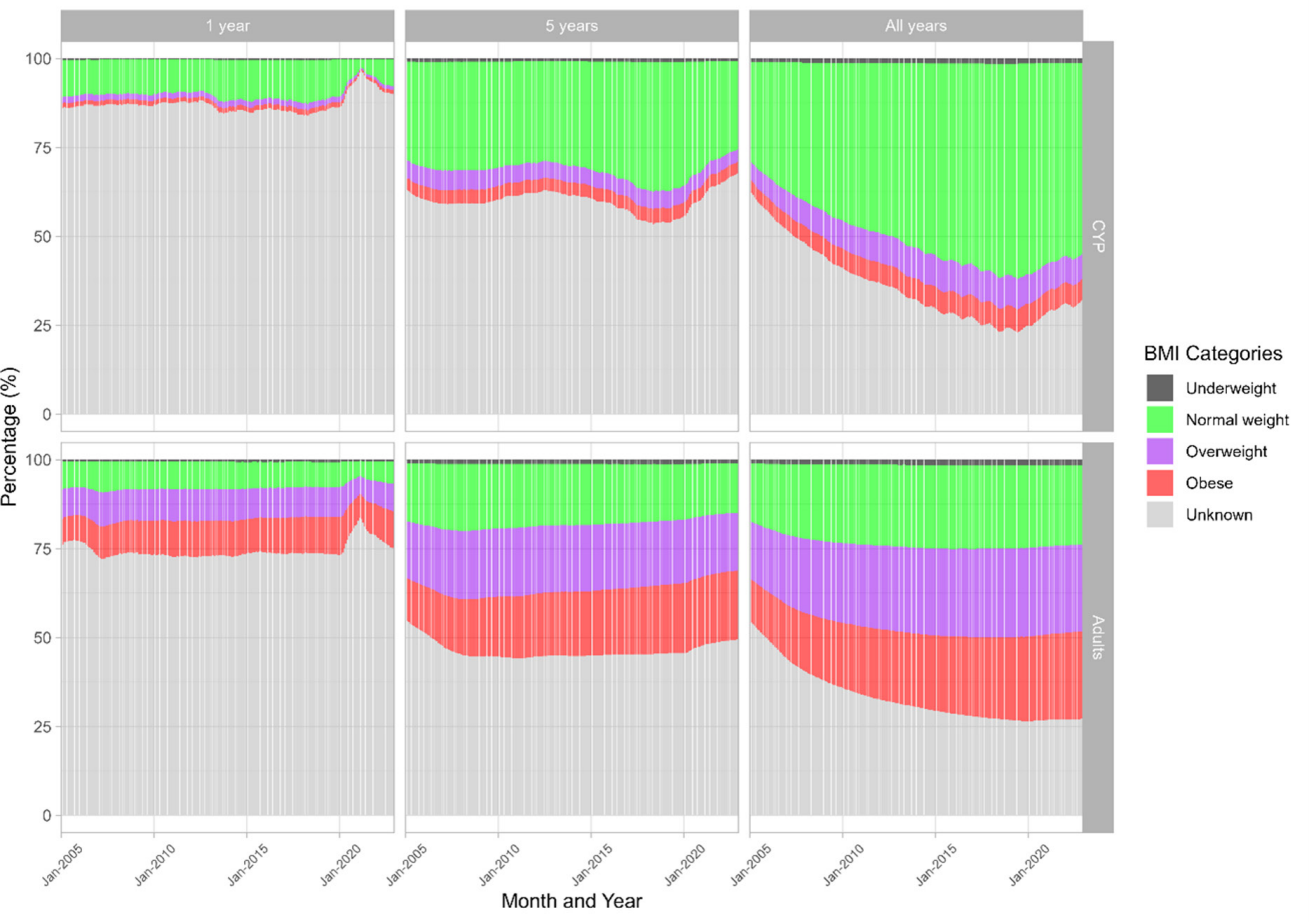
Impact of 1 year, 5 years and complete preservation of records on BMI record completeness. BMI, body mass index; CYP, children and young people.

Annually, a BMI record is documented for 10%–15% of the CYP population and 10%–26% of the adult population between 2000 and 2019. Both cohorts saw a decrease to 6% and 18% in 2020, followed by a steady increase (online supplemental material tables 3 and 4). The recapturing of information from the longitudinal datasets enabled us to update past observations. For preservation, the population was set to the population in December 2022 (647 711 CYP; 2 684 062 adults). Preserving BMI records yielded 10%, 33% and 68% for CYP and 25%, 51% and 73% for adults when preserving for 1 year, 5 years and across the study, respectively (figure 5).

## Discussion

We created a methodology that extracted and harmonised different data types relating to BMI from different data sources available within SAIL Databank to determine whether BMI coverage could be improved by incorporating more data sources and preserving longitudinal records of people living in Wales between 2000 and 2022.

The methodology showed that 56.2% of CYP and 65.2% of adult population in Wales have at least one BMI record between 2000 and 2022. In total, 964 049 (18.9%) unique individuals had their BMI recorded in at least 5 or more years across the study period, this includes 26 679 (3.0%) of CYP 937 370 (33.6%) of adults (see online supplemental material table 5). This is lower than the findings from England where about one-third of the population in England has a yearly BMI record in the primary care data,^8^ highlighting the need for more frequent recording of BMI components, particularly weight data, in primary care for individuals in Wales.

This methodology used four data sources to improve on previous studies where only one data source was used.^9 11 28^ For the CYP, incorporation of these data sources provided an additional 44% unique contribution from children’s community health data and 0.5% unique contribution from maternity records and hospital admissions. This demonstrated that having a data source that specifically collects BMI data is beneficial to increase coverage. For adults, 98% of BMI data came from general practice primary care, whereas hospital-based sources contributed little (<2%). These findings underscore the need to optimise data integration strategies, particularly for enhancing BMI capture beyond primary care settings which is the main source used in other studies.^9 11 28^ In other words, the recording of BMI-related data in settings outside the primary care, particularly in adults, could be improved to provide a more significant supplement to increase BMI coverage. Unique individuals from maternity records and hospital admissions accounted for less than 2%. This shows that unique individuals from maternity records and hospital admissions accounted for less than 2% of both cohorts. As the adult population did not have another data source dedicated to collecting BMI data, using data from primary care would be sufficient for BMI extraction. However, the community children’s data source (NCCH)^29^ provided a significant proportion of children’s BMI data supplementing the primary care source; therefore, only using primary care data would not be representative of the CYP population. We also note that the methodology applied same-day and over-time cleaning stages of cleaning for the adult cohort compared with only using same-day cleaning stage for the CYP cohort. This could account for the large difference in data from additional sources for the CYP cohort.

The methodology allowed comparison of the coverage of BMI data by preserving BMI readings recorded 1 or 5 years before the study end date and throughout the study period. We found an increase of 36% for CYP and 23% for adults when we preserved across the study period, compared with only preserving for 5 years, indicating that preserving BMI records increases coverage of BMI records. However,^30 30^ it was observed that older BMI records for individuals (3<years) tend to be on average 1.1 kg/m^2^ lower than more recent measurements, highlighting the potential for underestimation of BMI value when relying on historical records. While the study did not conclude that older BMI data are inherently unreliable, this finding underscores the need for caution when interpreting longitudinal BMI trends, particularly in adult populations. Additionally, children are likely to change due to developmental milestones; therefore, preserving a BMI record for a longer period may not be appropriate. This could also be the case for adults as changes could also happen during this period. However, as our methodology does not analyse the BMI data reliability, we cannot say whether preserving the BMI record for a longer time is a reliable way of accounting for the missingness, despite higher coverage. While BMI is not routinely used in children under 2 years according to Centers for Disease Control and Prevention guidance, the WHO provides validated growth standards that support its use in this age group, highlighting variation in practice and interpretation across international contexts.^31 32^

The methodology generated an RRDA with harmonised BMI categories for a population cohort representing the Welsh population. This is reproducible and customisable to different requirements and projects in different TREs. Therefore, one strength of the methodology is the practical application across research projects. Researchers could use the output generated using the population-level cohort or run the methodology on a smaller scale using their cohort of interest. Having an automated, standard script reduces errors that could arise from manually extracting and imputing data, allowing cross-checking of results. Furthermore, this could help design a more standardised approach to identifying and removing inconsistent data, leading to improved understanding and transparency regarding the limits of BMI or weight fluctuations in healthcare data, particularly for a longitudinal period. A key strength of our approach lies in the integration of multiple data sources, each differing in the frequency and context of BMI recording. This multi-source strategy helps mitigate some of the bias introduced by selective BMI measurement, particularly among individuals with greater healthcare utilisation. General practice data contributed the majority of adult BMI records, reflecting opportunistic recording during routine or condition-specific visits, while other data sources such as MIDS and NCCH were focused on specific programmes like maternity care and neonatal health, resulting in more structured and consistent BMI measurements across the population subgroups. To ensure consistency across diverse inputs, we applied a two-stage cleaning process: (a) same-day duplicates were ordered to flag implausible values as a pragmatic check and (b) longitudinal records were reviewed for extreme fluctuations. When multiple sources recorded BMIs on the same day, we retained values from the most reliable dataset according to a predefined hierarchy, though users may alternatively apply equal weighting. We also note that methods such as averaging, flagging or automated algorithms (eg, growthcleanr, Daymont *et al*^33^) represent valuable alternatives in certain contexts.

One limitation of this work is that the data sources used were specifically relevant to Wales and SAIL; therefore, the research asset produced currently is only available for Welsh data. However, we also provide a workflow that can be applied to other data sources of similar content and replicated in environments with similar BMI components and research studies.

Another limitation of our approach is that we only used obesity-related ICD-10 codes from hospital data. Other diagnoses that could be related to BMI are eating disorders, for example, anorexia, bulimia; however, these diagnoses do not have descriptions or guidelines for which BMI categories the individuals would fall into; hence, they were not included.

While this work focuses on improving the capture and harmonisation of BMI data, it is important to acknowledge the broader systemic issue: BMI measurement is not routinely prioritised across all sectors of the health system. Despite its known associations with a wide range of chronic conditions and its role in public health surveillance, BMI remains under-recorded in routine clinical settings. Only 10%–20% of the population in Wales had a BMI recorded during the 20-year study period. This reflects a deeper structural challenge: BMI is often recorded opportunistically, typically during clinical encounters where weight-related issues are already suspected, rather than as part of standardised, proactive monitoring. Factors contributing to this include time constraints in primary care, limited incentive structures, lack of integration between different healthcare services and an absence of clear national guidelines mandating regular BMI collection for all individuals (see online supplemental material figure 2). Moreover, there may be reluctance among healthcare professionals to raise weight-related topics without a clinical prompt, due to concerns about stigma or patient resistance. As such, our work is situated within this broader context of under-recording and seeks to address the methodological gap that results from these systemic practices. By demonstrating the added value of combining information held in various data sources and preserving longitudinal BMI records, our aim is not only to improve research-quality data, but also to inform future policy and infrastructure changes that could enable more systematic and equitable BMI monitoring at the population level.

Finally, the methodology is limited to the data in the available sources, reflecting on the limitations inherent to routinely collected EHR data, for example, BMI being missing from a large proportion of individuals. Contributing factors, such as height, are often not routinely recorded in adults. Although we address this by carrying forward the most recent height record for adults and pairing it with available weight data from all sources, it is important to note that adults may lose height as they age, which can introduce error into the carry-forward method if this is implemented on long time intervals.^34^ We acknowledge that this approach is not appropriate for children, as their height and BMI change rapidly and require time-specific measurements. It may be a clinical practice where individuals considered to have a healthy weight do not get their BMI data updated,^31^ thus contributing to the annual missingness rates. Although this may be a clinical approach, this will be considered missing data for each year that they are not recorded for research purposes, particularly if researchers who may use this methodology in the future decide to only preserve for shorter periods of time. It is important to note that the height and weight for CYP were routinely measured in the children’s community health data source and this cohort had a larger proportion of those with normal weight, whereas adults were more spread out across the categories, further highlighting the importance of routine recording of BMI across the population to have a more representative BMI data for the population.

Furthermore, Nicholson *et al*^8^ found that in England, individuals most likely to have repeated records of BMI were older individuals, females, those with higher BMI and people with clinical diagnoses. It was argued that this could create a bias where individuals with less pertinent clinical conditions may not be monitored as closely. Our data also showed that adult females have better coverage of BMI records than adult males, which means that our data are also biased towards certain groups of individuals. However, as our methodology did not incorporate clinical diagnosis with BMI records, we are not able to discern whether the repeated measurements are being driven by clinical relevance. This could be an avenue for future research. As most of our adult BMI-related information comes from primary care, it may be useful to record BMI information, even only weight data, more frequently from this resource regardless of health status, for example, at least every 2 years. Our findings also showed the minimal contribution from hospital-based sources (<2%), highlighting the need to improve BMI reporting in hospital data, as underlying electronic records likely capture more BMI measurements than currently reflected in structured datasets like PEDW for Wales and Hospital Episode Statistics (in England).

## Conclusion

This methodology improved previous methods of extracting BMI records from EHRs by incorporating four data sources for CYP and three data sources for adults. Incorporating additional data sources to generate a BMI RRDA has shown an improvement of 44% in BMI coverage for CYP, but less than 2% for adults.

Extending the retrieval period for the whole study period (2000–2022) has increased additional coverage by 35% for CYP and 23% for the adult cohort compared with only preserving for 5 years. Additionally, our methodology provides a reproducible approach for extracting and standardising BMI data types into BMI categories, increasing study efficiency for future research. However, it is important to note that retrieval of BMI records is pertinent to the quality and frequency of data recording in the first place.

### Key message

A significant proportion of individuals in Wales, UK, have unknown BMI. Our methodology leveraged anonymised, individual-level linked population-scale EHR data from the SAIL Databank, improving BMI coverage by 44% for children and young people (CYP but by less than 2% for the adults. Additionally, retaining BMI records over time further enhanced BMI coverage. This approach offers a reproducible, maintainable RRDA which can be used within TREs that contain similar linkable data sources with similar data types.

## Supplementary material

10.1136/bmjopen-2025-103724 online supplemental file 1

## Data Availability

This analysis uses the de-identified patient data accessed within the SAIL Databank trusted research environment and was approved by the SAIL independent Information Governance Review Panel (IGRP number: 0911). The IGRP gives careful consideration to each project to ensure proper and appropriate use of SAIL data, the current study uses double layer of anonymisation on patient data and therefore according the GDPR consent for publication deemed unnecessary. When access has been approved, it is gained through a privacy-protecting safe haven and remote access system referred to as the SAIL Gateway. SAIL has established an application process to be followed by anyone who would like to access data via SAIL https://www.saildatabank.com/application-process.
