# Supplementary material for: Bridging the gap: development of a methodology for retrieving and harmonising body mass index (BMI) from population-level linked electronic health records
Source: BMJ Open. 2025 Oct 5;15(10):e103724. doi: 10.1136/bmjopen-2025-103724 (PMC12506101; doi:10.1136/bmjopen-2025-103724)

Supplementary Material Table1. READ code list to extract BMI data in primary care data source.

| bmi_code | description | complexity | | category |
| --- | --- | --- | --- | --- |
| 2293 | O/E -height within 10% average | where event_val between x and y (depending on unit) | height | |
| 229.. | O/E - height | where event_val between x and y (depending on unit) | height | |
| 229Z. | O/E - height NOS | where event_val between x and y (depending on unit) | height | |
| 2292 | O/E - height 10-20% < average | height | height | |
| 2294 | O/E-height 10-20% over average | height | height | |
| 2295 | O/E -height > 20% over average | height | height | |
| 2291 | O/E-height > 20% below average | height | height | |
| 22A.. | O/E - weight | where event_val between 32 and 250 | weight | |
| 22A1. | O/E - weight > 20% below ideal | where event_val between 32 and 250 | weight | |
| 22A2. | O/E -weight 10-20% below ideal | where event_val between 32 and 250 | weight | |
| 22A3. | O/E - weight within 10% ideal | where event_val between 32 and 250 | weight | |
| 22A4. | O/E - weight 10-20% over ideal | where event_val between 32 and 250 | weight | |
| 22A5. | O/E - weight > 20% over ideal | where event_val between 32 and 250 | weight | |
| 22A6. | O/E - Underweight | where event_val between 32 and 250 | weight | |
| 22AA. | Overweight | where event_val between 32 and 250 | weight | |
| 22AZ. | O/E - weight NOS | where event_val between 32 and 250 | weight | |
| 1266 | FH: Obesity | Obese | obese | |
| 1444 | H/O: obesity | Obese | obese | |
| 22K3. | Body Mass Index low K/M2 | Underweight | underweight | |
| 22K.. | Body Mass Index | bmi | bmi | |
| 22K1. | Body Mass Index normal K/M2 | Normal weight | normal weight | |
| 22K2. | Body Mass Index high K/M2 | Overweight/Obese | obese | |
| 22K4. | Body mass index index 25-29 - overweight | Overweight | overweight | |
| 22K5. | Body mass index 30+ - obesity | Obese | obese | |
| 22K6. | Body mass index less than 20 | Underweight | underweight | |
| 22K7. | Body mass index 40+ - severely obese | Obese | obese | |
| 22K8. | Body mass index 20-24 - normal | Normal weight | normal weight | |
| 22K9. | Body mass index centile | bmi | bmi | |
| 22KC. | Obese class I (body mass index 30.0 - 34.9) | Obese | obese | |
| 22KC. | Obese class I (BMI 30.0-34.9) | Obese | obese | |
| 22KD. | Obese class II (body mass index 35.0 - 39.9) | Obese | obese | |
| 22KD. | Obese class II (BMI 35.0-39.9) | Obese | obese | |
| 22KE. | Obese class III (BMI equal to or greater than 40.0) | Obese | obese | |
| 22KE. | Obese cls III (BMI eq/gr 40.0) | Obese | obese | |
| 66C4. | Has seen dietician - obesity | Obese | obese | |
| 66C6. | Treatment of obesity started | Obese | obese | |
| 66CE. | Reason for obesity therapy - occupational | Obese | obese | |
| 8CV7. | Anti-obesity drug therapy commenced | Obese | obese | |
| 8T11. | Rfrrl multidisip obesity clin | Obese | obese | |
| C38.. | Obesity/oth hyperalimentation | Obese | obese | |
| C380. | Obesity | Obese | obese | |
| C3800 | Obesity due to excess calories | Obese | obese | |
| C3801 | Drug-induced obesity | Obese | obese | |
| C3802 | Extrem obesity+alveol hypovent | Obese | obese | |
| C3803 | Morbid obesity | Obese | obese | |
| C3804 | Central obesity | Obese | obese | |
| C3805 | Generalised obesity | Obese | obese | |
| C3806 | Adult-onset obesity | Obese | obese | |
| C3807 | Lifelong obesity | Obese | obese | |
| C38z. | Obesity/oth hyperalimentat NOS | Obese | obese | |
| C38z0 | Simple obesity NOS | Obese | obese | |
| Cyu7. | [X]Obesity+oth hyperalimentatn | Obese | obese | |
| 22K4. | BMI 25-29 - overweight | Overweight | overweight | |
| 22A1. | O/E - weight > 20% below ideal | Underweight | underweight | |
| 22A2. | O/E -weight 10-20% below ideal | Underweight | underweight | |
| 22A3. | O/E - weight within 10% ideal | Normal weight | normal weight | |
| 22A4. | O/E - weight 10-20% over ideal | Overweight | overweight | |
| 22A5. | O/E - weight > 20% over ideal | Overweight | overweight | |
| 22A6. | O/E - Underweight | Underweight | underweight | |
| 22AA. | Overweight | Overweight | overweight | |
| R0348 | [D] Underweight | Underweight | underweight | |
| 66C1. | Itinital obesity assessment | Obese | obese | |
| 66C2. | Follow-up obesity assessment | Obese | obese | |
| 66C5. | Treatment of obesity changed | Obese | obese | |
| 66CX. | Obesity multidisciplinary case review | Obese | obese | |
| 66CZ. | Obesity monitoring NOS | Obese | obese | |
| 9hN.. | Exception reporting: obesity quality indicators | Obese | obese | |
| 9OK.. | Obesity monitoring admin. | Obese | obese | |
| 9OK1. | Attends obesity monitoring | Obese | obese | |
| 9OK3. | Obesity monitoring default | Obese | obese | |
| 9OK2. | Refuses obesity monitoring | Obese | obese | |
| 9OK4. | Obesity monitoring 1st letter | Obese | obese | |
| 9OK5. | Obesity monitoring 2nd letter | Obese | obese | |
| 9OK6. | Obesity monitoring 3rd letter | Obese | obese | |
| 9OK7. | Obesity monitoring verbal inv. | Obese | obese | |
| 9OK8. | Obesity monitor phone invite | Obese | obese | |
| 9OKA. | Obesity monitoring check done | Obese | obese | |
| 9OKZ. | Obesity monitoring admin.NOS | Obese | obese | |
| C38y0 | Pickwickian syndrome | Obese | obese | |

Supplementary Material Table 2. ICD-10 code list to extract obesity related diagnoses

| icd_code | description | category |
| --- | --- | --- |
| E66.0 | Obesity due to excess calories | obese |
| E66.1 | Drug-induced obesity (use additional external cause code (Chapter XX, if desired, to identify the drug) | obese |
| E66.2 | Extreme obesity with alveolar hypoventilation (Obesity hypoventilation syndrome / Pickwickian syndrome) | obese |
| E66.8 | Other obesity (Morbid obesity) | obese |
| E66.9 | Obesity, unspecified (Simple obesity, NOS) | obese |

Supplementary Material Table 3. Yearly characteristics of the CYP cohort.

| **Characteristic** | **2000**  N = 689,458^1^ | **2001**  N = 686,346^1^ | **2002**  N = 685,317^1^ | **2003**  N = 683,107^1^ | **2004**  N = 679,517^1^ | **2005**  N = 680,863^1^ | **2006**  N = 676,446^1^ | **2007**  N = 671,645^1^ | **2008**  N = 666,298^1^ | **2009**  N = 660,510^1^ | **2010**  N = 655,864^1^ |
| --- | --- | --- | --- | --- | --- | --- | --- | --- | --- | --- | --- |
| **Sex** |  |  |  |  |  |  |  |  |  |  |  |
| Female | 337,873 (49%) | 336,208 (49%) | 335,539 (49%) | 334,135 (49%) | 332,176 (49%) | 332,956 (49%) | 330,563 (49%) | 327,959 (49%) | 325,348 (49%) | 322,569 (49%) | 320,126 (49%) |
| Male | 351,585 (51%) | 350,138 (51%) | 349,778 (51%) | 348,972 (51%) | 347,341 (51%) | 347,907 (51%) | 345,883 (51%) | 343,686 (51%) | 340,950 (51%) | 337,941 (51%) | 335,738 (51%) |
| **Ethnicity** |  |  |  |  |  |  |  |  |  |  |  |
| Asian | 10,418 (1.5%) | 10,964 (1.6%) | 11,746 (1.7%) | 12,859 (1.9%) | 13,861 (2.0%) | 14,908 (2.2%) | 16,207 (2.4%) | 17,516 (2.6%) | 18,618 (2.8%) | 19,568 (3.0%) | 20,458 (3.1%) |
| Black | 1,898 (0.3%) | 2,069 (0.3%) | 2,426 (0.4%) | 2,872 (0.4%) | 3,325 (0.5%) | 3,684 (0.5%) | 4,087 (0.6%) | 4,444 (0.7%) | 4,825 (0.7%) | 5,112 (0.8%) | 5,404 (0.8%) |
| Mixed | 6,904 (1.0%) | 7,270 (1.1%) | 7,711 (1.1%) | 8,158 (1.2%) | 8,606 (1.3%) | 9,098 (1.3%) | 9,613 (1.4%) | 10,148 (1.5%) | 10,649 (1.6%) | 11,242 (1.7%) | 11,795 (1.8%) |
| Other | 1,866 (0.3%) | 2,032 (0.3%) | 2,310 (0.3%) | 2,689 (0.4%) | 3,014 (0.4%) | 3,421 (0.5%) | 3,886 (0.6%) | 4,320 (0.6%) | 4,781 (0.7%) | 5,278 (0.8%) | 5,698 (0.9%) |
| Unknown | 53,288 (7.7%) | 45,760 (6.7%) | 37,966 (5.5%) | 29,537 (4.3%) | 23,942 (3.5%) | 20,155 (3.0%) | 15,535 (2.3%) | 11,810 (1.8%) | 9,448 (1.4%) | 7,694 (1.2%) | 6,310 (1.0%) |
| White | 615,084 (89%) | 618,251 (90%) | 623,158 (91%) | 626,992 (92%) | 626,769 (92%) | 629,597 (92%) | 627,118 (93%) | 623,407 (93%) | 617,977 (93%) | 611,616 (93%) | 606,199 (92%) |
| **Age band** |  |  |  |  |  |  |  |  |  |  |  |
| 2-5 | 135,688 (20%) | 132,464 (19%) | 128,280 (19%) | 125,377 (18%) | 120,581 (18%) | 124,006 (18%) | 124,699 (18%) | 126,266 (19%) | 129,985 (20%) | 131,858 (20%) | 134,897 (21%) |
| 5-13 | 316,688 (46%) | 313,535 (46%) | 311,834 (46%) | 308,511 (45%) | 305,946 (45%) | 301,813 (44%) | 295,830 (44%) | 290,828 (43%) | 284,996 (43%) | 281,895 (43%) | 277,411 (42%) |
| 13-19 | 237,082 (34%) | 240,347 (35%) | 245,203 (36%) | 249,219 (36%) | 252,990 (37%) | 255,044 (37%) | 255,917 (38%) | 254,551 (38%) | 251,317 (38%) | 246,757 (37%) | 243,556 (37%) |
| **BMI category** |  |  |  |  |  |  |  |  |  |  |  |
| Underweight | 1,565 (0.2%) | 1,683 (0.2%) | 1,751 (0.3%) | 1,887 (0.3%) | 2,205 (0.3%) | 2,031 (0.3%) | 1,980 (0.3%) | 1,874 (0.3%) | 1,818 (0.3%) | 1,804 (0.3%) | 1,720 (0.3%) |
| Normal weight | 40,881 (5.9%) | 45,841 (6.7%) | 53,731 (7.8%) | 56,649 (8.3%) | 69,772 (10%) | 65,838 (9.7%) | 65,341 (9.7%) | 63,447 (9.4%) | 62,372 (9.4%) | 64,520 (9.8%) | 59,772 (9.1%) |
| Obese | 4,514 (0.7%) | 5,173 (0.8%) | 6,186 (0.9%) | 6,881 (1.0%) | 9,041 (1.3%) | 8,583 (1.3%) | 8,540 (1.3%) | 8,513 (1.3%) | 8,646 (1.3%) | 8,715 (1.3%) | 8,304 (1.3%) |
| Overweight | 7,109 (1.0%) | 7,952 (1.2%) | 9,290 (1.4%) | 10,026 (1.5%) | 12,421 (1.8%) | 11,753 (1.7%) | 11,363 (1.7%) | 10,843 (1.6%) | 10,778 (1.6%) | 10,960 (1.7%) | 10,186 (1.6%) |
| Unknown | 635,389 (92%) | 625,697 (91%) | 614,359 (90%) | 607,664 (89%) | 586,078 (86%) | 592,658 (87%) | 589,222 (87%) | 586,968 (87%) | 582,684 (87%) | 574,511 (87%) | 575,882 (88%) |
| **Welsh Index of Multiple Deprivation 2019*** |  |  |  |  |  |  |  |  |  |  |  |
| 1. Most deprived | 159,543 (23%) | 157,819 (23%) | 156,548 (23%) | 155,242 (23%) | 154,014 (23%) | 153,819 (23%) | 152,531 (23%) | 150,847 (22%) | 149,766 (22%) | 147,843 (22%) | 146,998 (22%) |
| 2 | 139,840 (20%) | 138,743 (20%) | 138,271 (20%) | 137,447 (20%) | 136,388 (20%) | 135,998 (20%) | 134,978 (20%) | 133,845 (20%) | 132,669 (20%) | 131,607 (20%) | 130,794 (20%) |
| 3 | 133,159 (19%) | 132,200 (19%) | 131,739 (19%) | 131,449 (19%) | 130,790 (19%) | 130,814 (19%) | 129,731 (19%) | 129,002 (19%) | 127,972 (19%) | 126,666 (19%) | 125,475 (19%) |
| 4 | 128,755 (19%) | 128,422 (19%) | 128,584 (19%) | 128,598 (19%) | 128,275 (19%) | 129,089 (19%) | 128,381 (19%) | 127,595 (19%) | 126,307 (19%) | 125,184 (19%) | 124,037 (19%) |
| 5. Least deprived | 128,161 (19%) | 129,162 (19%) | 130,175 (19%) | 130,371 (19%) | 130,050 (19%) | 131,143 (19%) | 130,825 (19%) | 130,356 (19%) | 129,584 (19%) | 129,210 (20%) | 128,560 (20%) |
| **Rural urban classification** |  |  |  |  |  |  |  |  |  |  |  |
| Rural town and fringe | 93,720 (14%) | 93,230 (14%) | 92,772 (14%) | 92,580 (14%) | 92,149 (14%) | 92,377 (14%) | 91,715 (14%) | 90,771 (14%) | 90,001 (14%) | 89,107 (13%) | 88,221 (13%) |
| Rural town and fringe in a sparse setting | 26,219 (3.8%) | 26,011 (3.8%) | 26,099 (3.8%) | 26,189 (3.8%) | 25,991 (3.8%) | 26,198 (3.8%) | 25,912 (3.8%) | 25,595 (3.8%) | 25,227 (3.8%) | 24,803 (3.8%) | 24,495 (3.7%) |
| Rural village and dispersed | 44,600 (6.5%) | 44,649 (6.5%) | 44,754 (6.5%) | 44,662 (6.5%) | 44,683 (6.6%) | 44,871 (6.6%) | 44,377 (6.6%) | 43,970 (6.5%) | 43,528 (6.5%) | 42,915 (6.5%) | 42,347 (6.5%) |
| Rural village and dispersed in a sparse setting | 49,149 (7.1%) | 48,880 (7.1%) | 48,850 (7.1%) | 48,872 (7.2%) | 48,779 (7.2%) | 48,987 (7.2%) | 48,622 (7.2%) | 48,414 (7.2%) | 47,721 (7.2%) | 47,004 (7.1%) | 46,201 (7.0%) |
| Urban city and town | 462,838 (67%) | 460,646 (67%) | 459,787 (67%) | 457,713 (67%) | 455,185 (67%) | 455,639 (67%) | 453,016 (67%) | 450,346 (67%) | 447,295 (67%) | 444,191 (67%) | 441,949 (67%) |
| Urban city and town in a sparse setting | 12,932 (1.9%) | 12,930 (1.9%) | 13,055 (1.9%) | 13,091 (1.9%) | 12,730 (1.9%) | 12,791 (1.9%) | 12,804 (1.9%) | 12,549 (1.9%) | 12,526 (1.9%) | 12,490 (1.9%) | 12,651 (1.9%) |
| ^1^n (%) | | | | | | | | | | | |

Supplementary Material Table 3. Yearly characteristics of the CYP cohort (continued).

| **Characteristic** | **2011**  N = 652,066^1^ | **2012**  N = 650,498^1^ | **2013**  N = 648,913^1^ | **2014**  N = 648,596^1^ | **2015**  N = 646,871^1^ | **2016**  N = 646,678^1^ | **2017**  N = 645,702^1^ | **2018**  N = 644,727^1^ | **2019**  N = 645,187^1^ | **2020**  N = 640,918^1^ | **2021**  N = 641,458^1^ | **2022**  N = 647,711^1^ |
| --- | --- | --- | --- | --- | --- | --- | --- | --- | --- | --- | --- | --- |
| **Sex** |  |  |  |  |  |  |  |  |  |  |  |  |
| Female | 318,139 (49%) | 317,237 (49%) | 316,647 (49%) | 316,332 (49%) | 315,444 (49%) | 315,393 (49%) | 314,896 (49%) | 314,390 (49%) | 314,722 (49%) | 312,983 (49%) | 313,399 (49%) | 316,638 (49%) |
| Male | 333,927 (51%) | 333,261 (51%) | 332,266 (51%) | 332,264 (51%) | 331,427 (51%) | 331,285 (51%) | 330,806 (51%) | 330,337 (51%) | 330,465 (51%) | 327,935 (51%) | 328,059 (51%) | 331,073 (51%) |
| **Ethnicity** |  |  |  |  |  |  |  |  |  |  |  |  |
| Asian | 21,165 (3.2%) | 21,851 (3.4%) | 22,286 (3.4%) | 22,752 (3.5%) | 23,098 (3.6%) | 23,633 (3.7%) | 24,208 (3.7%) | 24,589 (3.8%) | 25,156 (3.9%) | 25,255 (3.9%) | 25,761 (4.0%) | 25,062 (3.9%) |
| Black | 5,721 (0.9%) | 5,932 (0.9%) | 6,107 (0.9%) | 6,398 (1.0%) | 6,607 (1.0%) | 6,791 (1.1%) | 6,959 (1.1%) | 7,189 (1.1%) | 7,493 (1.2%) | 7,477 (1.2%) | 7,743 (1.2%) | 7,544 (1.2%) |
| Mixed | 12,412 (1.9%) | 13,094 (2.0%) | 13,794 (2.1%) | 14,642 (2.3%) | 15,269 (2.4%) | 16,100 (2.5%) | 16,893 (2.6%) | 17,682 (2.7%) | 18,273 (2.8%) | 18,483 (2.9%) | 18,840 (2.9%) | 18,727 (2.9%) |
| Other | 6,022 (0.9%) | 6,343 (1.0%) | 6,787 (1.0%) | 7,158 (1.1%) | 7,402 (1.1%) | 7,888 (1.2%) | 8,329 (1.3%) | 8,672 (1.3%) | 9,061 (1.4%) | 8,995 (1.4%) | 8,910 (1.4%) | 8,569 (1.3%) |
| Unknown | 6,336 (1.0%) | 7,656 (1.2%) | 7,695 (1.2%) | 7,400 (1.1%) | 7,372 (1.1%) | 7,856 (1.2%) | 7,717 (1.2%) | 7,670 (1.2%) | 9,409 (1.5%) | 11,518 (1.8%) | 17,153 (2.7%) | 35,841 (5.5%) |
| White | 600,410 (92%) | 595,622 (92%) | 592,244 (91%) | 590,246 (91%) | 587,123 (91%) | 584,410 (90%) | 581,596 (90%) | 578,925 (90%) | 575,795 (89%) | 569,190 (89%) | 563,051 (88%) | 551,968 (85%) |
| **Age band** |  |  |  |  |  |  |  |  |  |  |  |  |
| 2-5 | 136,972 (21%) | 139,391 (21%) | 136,832 (21%) | 135,969 (21%) | 136,744 (21%) | 134,080 (21%) | 132,115 (20%) | 130,348 (20%) | 128,757 (20%) | 131,352 (20%) | 125,085 (20%) | 121,173 (19%) |
| 5-13 | 274,831 (42%) | 274,120 (42%) | 280,038 (43%) | 284,669 (44%) | 286,094 (44%) | 293,283 (45%) | 297,689 (46%) | 300,420 (47%) | 302,281 (47%) | 296,625 (46%) | 299,142 (47%) | 302,157 (47%) |
| 13-18 | 240,263 (37%) | 236,987 (36%) | 232,043 (36%) | 227,958 (35%) | 224,033 (35%) | 219,315 (34%) | 215,898 (33%) | 213,959 (33%) | 214,149 (33%) | 212,941 (33%) | 217,231 (34%) | 224,381 (35%) |
| **BMI category** |  |  |  |  |  |  |  |  |  |  |  |  |
| Underweight | 1,757 (0.3%) | 1,793 (0.3%) | 1,994 (0.3%) | 1,950 (0.3%) | 2,070 (0.3%) | 2,054 (0.3%) | 2,328 (0.4%) | 2,246 (0.3%) | 1,816 (0.3%) | 973 (0.2%) | 1,212 (0.2%) | 1,667 (0.3%) |
| Normal weight | 59,453 (9.1%) | 60,383 (9.3%) | 73,883 (11%) | 72,175 (11%) | 68,303 (11%) | 70,402 (11%) | 75,082 (12%) | 71,110 (11%) | 65,880 (10%) | 28,096 (4.4%) | 32,064 (5.0%) | 47,870 (7.4%) |
| Obese | 8,260 (1.3%) | 8,129 (1.2%) | 9,042 (1.4%) | 8,776 (1.4%) | 8,552 (1.3%) | 9,122 (1.4%) | 9,491 (1.5%) | 8,886 (1.4%) | 8,896 (1.4%) | 4,406 (0.7%) | 5,845 (0.9%) | 7,157 (1.1%) |
| Overweight | 9,928 (1.5%) | 9,894 (1.5%) | 11,311 (1.7%) | 10,508 (1.6%) | 10,194 (1.6%) | 10,460 (1.6%) | 11,195 (1.7%) | 10,515 (1.6%) | 9,853 (1.5%) | 4,342 (0.7%) | 5,243 (0.8%) | 6,815 (1.1%) |
| Unknown | 572,668 (88%) | 570,299 (88%) | 552,683 (85%) | 555,187 (86%) | 557,752 (86%) | 554,640 (86%) | 547,606 (85%) | 551,970 (86%) | 558,742 (87%) | 603,101 (94%) | 597,094 (93%) | 584,202 (90%) |
| **Welsh Index of Multiple Deprivation 2019*** |  |  |  |  |  |  |  |  |  |  |  |  |
| 1. Most deprived | 146,247 (22%) | 146,397 (23%) | 147,243 (23%) | 148,257 (23%) | 148,967 (23%) | 149,732 (23%) | 150,513 (23%) | 151,096 (23%) | 151,878 (24%) | 151,688 (24%) | 151,621 (24%) | 152,971 (24%) |
| 2 | 130,000 (20%) | 129,909 (20%) | 129,724 (20%) | 129,585 (20%) | 129,124 (20%) | 129,097 (20%) | 129,059 (20%) | 128,736 (20%) | 128,998 (20%) | 128,201 (20%) | 128,124 (20%) | 128,797 (20%) |
| 3 | 124,646 (19%) | 124,700 (19%) | 124,070 (19%) | 123,926 (19%) | 123,621 (19%) | 123,594 (19%) | 123,220 (19%) | 122,953 (19%) | 123,114 (19%) | 121,875 (19%) | 122,404 (19%) | 123,781 (19%) |
| 4 | 123,028 (19%) | 122,517 (19%) | 121,847 (19%) | 121,471 (19%) | 121,101 (19%) | 120,833 (19%) | 120,460 (19%) | 120,187 (19%) | 119,755 (19%) | 119,443 (19%) | 119,536 (19%) | 120,644 (19%) |
| 5. Least deprived | 128,145 (20%) | 126,975 (20%) | 126,029 (19%) | 125,357 (19%) | 124,058 (19%) | 123,422 (19%) | 122,450 (19%) | 121,755 (19%) | 121,442 (19%) | 119,711 (19%) | 119,773 (19%) | 121,518 (19%) |
| **Rural urban classification** |  |  |  |  |  |  |  |  |  |  |  |  |
| Rural town and fringe | 87,508 (13%) | 87,048 (13%) | 86,409 (13%) | 86,173 (13%) | 85,801 (13%) | 85,535 (13%) | 85,476 (13%) | 85,115 (13%) | 84,995 (13%) | 84,726 (13%) | 84,669 (13%) | 85,292 (13%) |
| Rural town and fringe in a sparse setting | 24,278 (3.7%) | 23,955 (3.7%) | 23,602 (3.6%) | 23,441 (3.6%) | 23,152 (3.6%) | 22,904 (3.5%) | 22,659 (3.5%) | 22,510 (3.5%) | 22,578 (3.5%) | 22,339 (3.5%) | 22,269 (3.5%) | 22,254 (3.4%) |
| Rural village and dispersed | 41,722 (6.4%) | 41,315 (6.4%) | 40,800 (6.3%) | 40,400 (6.2%) | 40,029 (6.2%) | 39,923 (6.2%) | 39,577 (6.1%) | 39,231 (6.1%) | 39,077 (6.1%) | 38,754 (6.0%) | 38,844 (6.1%) | 38,951 (6.0%) |
| Rural village and dispersed in a sparse setting | 45,341 (7.0%) | 44,891 (6.9%) | 44,502 (6.9%) | 43,935 (6.8%) | 43,416 (6.7%) | 42,991 (6.6%) | 42,413 (6.6%) | 42,116 (6.5%) | 41,743 (6.5%) | 41,583 (6.5%) | 41,654 (6.5%) | 41,740 (6.4%) |
| Urban city and town | 440,450 (68%) | 440,466 (68%) | 441,053 (68%) | 442,262 (68%) | 442,284 (68%) | 443,214 (69%) | 443,425 (69%) | 443,508 (69%) | 444,713 (69%) | 441,478 (69%) | 442,007 (69%) | 447,096 (69%) |
| Urban city and town in a sparse setting | 12,767 (2.0%) | 12,823 (2.0%) | 12,547 (1.9%) | 12,385 (1.9%) | 12,189 (1.9%) | 12,111 (1.9%) | 12,152 (1.9%) | 12,247 (1.9%) | 12,081 (1.9%) | 12,038 (1.9%) | 12,015 (1.9%) | 12,378 (1.9%) |
| ^1^n (%) | | | | | | | | | | | | |

Supplementary Material Table 4. Yearly characteristics of the adult cohort

| **Characteristic** | **2000**  N = 2,366,129^1^ | **2001**  N = 2,382,514^1^ | **2002**  N = 2,402,849^1^ | **2003**  N = 2,423,442^1^ | **2004**  N = 2,452,682^1^ | **2005**  N = 2,477,928^1^ | **2006**  N = 2,496,932^1^ | **2007**  N = 2,518,536^1^ | **2008**  N = 2,532,634^1^ | **2009**  N = 2,544,351^1^ | **2010**  N = 2,555,407^1^ |
| --- | --- | --- | --- | --- | --- | --- | --- | --- | --- | --- | --- |
| **Sex** |  |  |  |  |  |  |  |  |  |  |  |
| Female | 1,210,454 (51%) | 1,216,972 (51%) | 1,226,562 (51%) | 1,236,767 (51%) | 1,249,489 (51%) | 1,260,598 (51%) | 1,268,670 (51%) | 1,277,743 (51%) | 1,282,778 (51%) | 1,285,998 (51%) | 1,289,885 (50%) |
| Male | 1,155,675 (49%) | 1,165,542 (49%) | 1,176,287 (49%) | 1,186,675 (49%) | 1,203,193 (49%) | 1,217,330 (49%) | 1,228,262 (49%) | 1,240,793 (49%) | 1,249,856 (49%) | 1,258,353 (49%) | 1,265,522 (50%) |
| **Ethnicity** |  |  |  |  |  |  |  |  |  |  |  |
| Asian | 20,513 (0.9%) | 22,260 (0.9%) | 23,993 (1.0%) | 26,398 (1.1%) | 29,322 (1.2%) | 32,546 (1.3%) | 37,305 (1.5%) | 42,731 (1.7%) | 47,639 (1.9%) | 51,943 (2.0%) | 56,534 (2.2%) |
| Black | 4,748 (0.2%) | 5,086 (0.2%) | 5,619 (0.2%) | 6,275 (0.3%) | 7,044 (0.3%) | 7,992 (0.3%) | 9,177 (0.4%) | 10,418 (0.4%) | 11,367 (0.4%) | 12,352 (0.5%) | 13,190 (0.5%) |
| Mixed | 6,377 (0.3%) | 6,800 (0.3%) | 7,366 (0.3%) | 8,021 (0.3%) | 8,751 (0.4%) | 9,548 (0.4%) | 10,517 (0.4%) | 11,645 (0.5%) | 12,559 (0.5%) | 13,449 (0.5%) | 14,257 (0.6%) |
| Other | 4,411 (0.2%) | 4,734 (0.2%) | 5,227 (0.2%) | 5,802 (0.2%) | 6,516 (0.3%) | 7,448 (0.3%) | 8,888 (0.4%) | 10,587 (0.4%) | 12,062 (0.5%) | 13,527 (0.5%) | 14,932 (0.6%) |
| Unknown | 474,451 (20%) | 444,313 (19%) | 415,408 (17%) | 383,858 (16%) | 357,252 (15%) | 326,713 (13%) | 284,841 (11%) | 242,439 (9.6%) | 204,651 (8.1%) | 173,472 (6.8%) | 145,859 (5.7%) |
| White | 1,855,629 (78%) | 1,899,321 (80%) | 1,945,236 (81%) | 1,993,088 (82%) | 2,043,797 (83%) | 2,093,681 (84%) | 2,146,204 (86%) | 2,200,716 (87%) | 2,244,356 (89%) | 2,279,608 (90%) | 2,310,635 (90%) |
| **Age band** |  |  |  |  |  |  |  |  |  |  |  |
| 19-29 | 446,263 (19%) | 442,694 (19%) | 443,078 (18%) | 446,133 (18%) | 455,983 (19%) | 466,681 (19%) | 475,530 (19%) | 486,525 (19%) | 493,428 (19%) | 495,825 (19%) | 496,668 (19%) |
| 30-39 | 451,042 (19%) | 452,818 (19%) | 450,948 (19%) | 446,432 (18%) | 441,556 (18%) | 434,968 (18%) | 426,771 (17%) | 418,341 (17%) | 409,232 (16%) | 404,446 (16%) | 400,311 (16%) |
| 40-49 | 399,555 (17%) | 405,630 (17%) | 414,174 (17%) | 422,669 (17%) | 434,004 (18%) | 444,733 (18%) | 451,194 (18%) | 456,755 (18%) | 461,676 (18%) | 463,813 (18%) | 463,208 (18%) |
| 50-59 | 387,694 (16%) | 396,373 (17%) | 401,780 (17%) | 406,370 (17%) | 407,957 (17%) | 410,162 (17%) | 407,884 (16%) | 401,810 (16%) | 399,689 (16%) | 399,826 (16%) | 403,274 (16%) |
| 60-69 | 292,543 (12%) | 295,007 (12%) | 301,495 (13%) | 309,744 (13%) | 318,738 (13%) | 325,018 (13%) | 337,082 (13%) | 352,594 (14%) | 362,659 (14%) | 370,772 (15%) | 377,293 (15%) |
| 70-79 | 241,995 (10%) | 238,306 (10%) | 236,920 (9.9%) | 234,223 (9.7%) | 234,808 (9.6%) | 234,777 (9.5%) | 235,341 (9.4%) | 237,920 (9.4%) | 239,984 (9.5%) | 242,602 (9.5%) | 245,346 (9.6%) |
| 80-89 | 121,877 (5.2%) | 125,976 (5.3%) | 127,812 (5.3%) | 130,556 (5.4%) | 131,752 (5.4%) | 133,648 (5.4%) | 135,096 (5.4%) | 136,547 (5.4%) | 137,920 (5.4%) | 138,179 (5.4%) | 137,705 (5.4%) |
| 90 and over | 25,160 (1.1%) | 25,710 (1.1%) | 26,642 (1.1%) | 27,315 (1.1%) | 27,884 (1.1%) | 27,941 (1.1%) | 28,034 (1.1%) | 28,044 (1.1%) | 28,046 (1.1%) | 28,888 (1.1%) | 31,602 (1.2%) |
| **BMI category** |  |  |  |  |  |  |  |  |  |  |  |
| Underweight | 5,374 (0.2%) | 6,029 (0.3%) | 7,239 (0.3%) | 10,160 (0.4%) | 12,799 (0.5%) | 12,532 (0.5%) | 14,439 (0.6%) | 14,491 (0.6%) | 13,596 (0.5%) | 14,921 (0.6%) | 14,843 (0.6%) |
| Normal weight | 81,776 (3.5%) | 92,512 (3.9%) | 108,254 (4.5%) | 155,479 (6.4%) | 194,852 (7.9%) | 176,117 (7.1%) | 205,152 (8.2%) | 202,403 (8.0%) | 182,851 (7.2%) | 195,218 (7.7%) | 193,938 (7.6%) |
| Obese | 59,532 (2.5%) | 77,642 (3.3%) | 98,926 (4.1%) | 134,913 (5.6%) | 170,869 (7.0%) | 172,997 (7.0%) | 213,257 (8.5%) | 222,971 (8.9%) | 234,310 (9.3%) | 238,233 (9.4%) | 245,842 (9.6%) |
| Overweight | 76,857 (3.2%) | 92,200 (3.9%) | 114,081 (4.7%) | 161,984 (6.7%) | 205,004 (8.4%) | 190,406 (7.7%) | 226,281 (9.1%) | 226,513 (9.0%) | 216,320 (8.5%) | 222,586 (8.7%) | 224,569 (8.8%) |
| Unknown | 2,142,590 (91%) | 2,114,131 (89%) | 2,074,349 (86%) | 1,960,906 (81%) | 1,869,158 (76%) | 1,925,876 (78%) | 1,837,803 (74%) | 1,852,158 (74%) | 1,885,557 (74%) | 1,873,393 (74%) | 1,876,215 (73%) |
| **Welsh Index of Multiple Deprivation 2019*** |  |  |  |  |  |  |  |  |  |  |  |
| 1. Most deprived | 460,171 (19%) | 460,445 (19%) | 461,757 (19%) | 464,043 (19%) | 468,379 (19%) | 472,977 (19%) | 476,463 (19%) | 480,152 (19%) | 481,581 (19%) | 483,930 (19%) | 485,980 (19%) |
| 2 | 469,615 (20%) | 470,993 (20%) | 473,448 (20%) | 476,251 (20%) | 481,386 (20%) | 486,230 (20%) | 490,305 (20%) | 494,238 (20%) | 497,646 (20%) | 499,548 (20%) | 501,672 (20%) |
| 3 | 493,498 (21%) | 497,570 (21%) | 501,408 (21%) | 505,512 (21%) | 512,812 (21%) | 518,260 (21%) | 521,854 (21%) | 527,063 (21%) | 529,642 (21%) | 532,193 (21%) | 535,165 (21%) |
| 4 | 475,080 (20%) | 478,778 (20%) | 484,201 (20%) | 490,267 (20%) | 496,696 (20%) | 502,471 (20%) | 506,534 (20%) | 511,227 (20%) | 515,011 (20%) | 517,015 (20%) | 518,791 (20%) |
| 5. Least deprived | 467,765 (20%) | 474,728 (20%) | 482,035 (20%) | 487,369 (20%) | 493,409 (20%) | 497,990 (20%) | 501,776 (20%) | 505,856 (20%) | 508,754 (20%) | 511,665 (20%) | 513,799 (20%) |
| **Rural urban classification** |  |  |  |  |  |  |  |  |  |  |  |
| Rural town and fringe | 311,443 (13%) | 312,659 (13%) | 314,414 (13%) | 317,539 (13%) | 321,305 (13%) | 324,159 (13%) | 326,347 (13%) | 328,624 (13%) | 330,270 (13%) | 331,571 (13%) | 332,760 (13%) |
| Rural town and fringe in a sparse setting | 97,271 (4.1%) | 97,362 (4.1%) | 97,969 (4.1%) | 99,337 (4.1%) | 100,444 (4.1%) | 101,133 (4.1%) | 101,713 (4.1%) | 102,554 (4.1%) | 102,589 (4.1%) | 102,779 (4.0%) | 102,582 (4.0%) |
| Rural village and dispersed | 162,114 (6.9%) | 163,331 (6.9%) | 165,484 (6.9%) | 167,353 (6.9%) | 169,702 (6.9%) | 171,740 (6.9%) | 172,682 (6.9%) | 173,987 (6.9%) | 174,350 (6.9%) | 174,752 (6.9%) | 174,868 (6.8%) |
| Rural village and dispersed in a sparse setting | 181,049 (7.7%) | 182,576 (7.7%) | 185,156 (7.7%) | 187,875 (7.8%) | 190,840 (7.8%) | 192,745 (7.8%) | 194,028 (7.8%) | 195,912 (7.8%) | 196,416 (7.8%) | 197,328 (7.8%) | 197,677 (7.7%) |
| Urban city and town | 1,565,691 (66%) | 1,577,594 (66%) | 1,590,217 (66%) | 1,600,995 (66%) | 1,619,525 (66%) | 1,636,856 (66%) | 1,650,587 (66%) | 1,665,736 (66%) | 1,677,294 (66%) | 1,685,919 (66%) | 1,695,242 (66%) |
| Urban city and town in a sparse setting | 48,561 (2.1%) | 48,992 (2.1%) | 49,609 (2.1%) | 50,343 (2.1%) | 50,866 (2.1%) | 51,295 (2.1%) | 51,575 (2.1%) | 51,723 (2.1%) | 51,715 (2.0%) | 52,002 (2.0%) | 52,278 (2.0%) |
|  |  | ^1^n (%) | | | | | | | | | |

Supplementary Material Table 4. Yearly characteristics of the adult cohort (continued)

| **Characteristic** | **2011**  N = 2,561,957^1^ | **2012**  N = 2,576,621^1^ | **2013**  N = 2,590,054^1^ | **2014**  N = 2,596,473^1^ | **2015**  N = 2,606,774^1^ | **2016**  N = 2,619,908^1^ | **2017**  N = 2,631,428^1^ | **2018**  N = 2,639,575^1^ | **2019**  N = 2,649,185^1^ | **2020**  N = 2,646,163^1^ | **2021**  N = 2,677,342^1^ | **2022**  N = 2,684,062^1^ |
| --- | --- | --- | --- | --- | --- | --- | --- | --- | --- | --- | --- | --- |
| **Sex** |  |  |  |  |  |  |  |  |  |  |  |  |
| Female | 1,292,262 (50%) | 1,299,572 (50%) | 1,305,165 (50%) | 1,309,289 (50%) | 1,314,083 (50%) | 1,319,674 (50%) | 1,326,011 (50%) | 1,331,372 (50%) | 1,335,966 (50%) | 1,334,496 (50%) | 1,347,900 (50%) | 1,354,562 (50%) |
| Male | 1,269,695 (50%) | 1,277,049 (50%) | 1,284,889 (50%) | 1,287,184 (50%) | 1,292,691 (50%) | 1,300,234 (50%) | 1,305,417 (50%) | 1,308,203 (50%) | 1,313,219 (50%) | 1,311,667 (50%) | 1,329,442 (50%) | 1,329,500 (50%) |
| **Ethnicity** |  |  |  |  |  |  |  |  |  |  |  |  |
| Asian | 59,317 (2.3%) | 60,929 (2.4%) | 60,950 (2.4%) | 62,213 (2.4%) | 64,224 (2.5%) | 65,408 (2.5%) | 66,296 (2.5%) | 67,395 (2.6%) | 70,392 (2.7%) | 72,681 (2.7%) | 79,202 (3.0%) | 75,808 (2.8%) |
| Black | 13,938 (0.5%) | 14,476 (0.6%) | 14,851 (0.6%) | 15,388 (0.6%) | 16,073 (0.6%) | 16,667 (0.6%) | 17,283 (0.7%) | 17,838 (0.7%) | 18,776 (0.7%) | 19,555 (0.7%) | 21,609 (0.8%) | 21,134 (0.8%) |
| Mixed | 14,998 (0.6%) | 15,685 (0.6%) | 16,236 (0.6%) | 16,875 (0.6%) | 17,569 (0.7%) | 18,352 (0.7%) | 18,971 (0.7%) | 19,775 (0.7%) | 20,664 (0.8%) | 21,384 (0.8%) | 22,598 (0.8%) | 22,446 (0.8%) |
| Other | 15,988 (0.6%) | 16,951 (0.7%) | 17,922 (0.7%) | 18,925 (0.7%) | 19,788 (0.8%) | 20,987 (0.8%) | 22,241 (0.8%) | 23,326 (0.9%) | 24,735 (0.9%) | 25,652 (1.0%) | 27,620 (1.0%) | 26,857 (1.0%) |
| Unknown | 123,524 (4.8%) | 118,548 (4.6%) | 120,777 (4.7%) | 118,553 (4.6%) | 116,521 (4.5%) | 115,894 (4.4%) | 114,729 (4.4%) | 112,754 (4.3%) | 110,496 (4.2%) | 102,144 (3.9%) | 105,635 (3.9%) | 146,554 (5.5%) |
| White | 2,334,192 (91%) | 2,350,032 (91%) | 2,359,318 (91%) | 2,364,519 (91%) | 2,372,599 (91%) | 2,382,600 (91%) | 2,391,908 (91%) | 2,398,487 (91%) | 2,404,122 (91%) | 2,404,747 (91%) | 2,420,678 (90%) | 2,391,263 (89%) |
| **Age band** |  |  |  |  |  |  |  |  |  |  |  |  |
| 19-29 | 498,189 (19%) | 500,304 (19%) | 500,559 (19%) | 495,236 (19%) | 491,860 (19%) | 489,282 (19%) | 483,954 (18%) | 476,236 (18%) | 469,739 (18%) | 453,721 (17%) | 455,371 (17%) | 445,494 (17%) |
| 30-39 | 393,622 (15%) | 392,686 (15%) | 393,470 (15%) | 395,779 (15%) | 401,316 (15%) | 407,961 (16%) | 416,113 (16%) | 422,663 (16%) | 425,755 (16%) | 427,618 (16%) | 437,945 (16%) | 444,584 (17%) |
| 40-49 | 462,106 (18%) | 457,537 (18%) | 451,513 (17%) | 442,009 (17%) | 431,676 (17%) | 422,919 (16%) | 413,690 (16%) | 404,496 (15%) | 400,882 (15%) | 397,442 (15%) | 396,878 (15%) | 397,832 (15%) |
| 50-59 | 407,483 (16%) | 415,018 (16%) | 422,638 (16%) | 431,742 (17%) | 441,089 (17%) | 447,365 (17%) | 452,973 (17%) | 458,418 (17%) | 462,616 (17%) | 462,976 (17%) | 467,510 (17%) | 463,004 (17%) |
| 60-69 | 382,403 (15%) | 384,893 (15%) | 387,868 (15%) | 387,663 (15%) | 389,080 (15%) | 387,383 (15%) | 382,993 (15%) | 382,903 (15%) | 384,974 (15%) | 389,418 (15%) | 397,575 (15%) | 405,700 (15%) |
| 70-79 | 247,535 (9.7%) | 253,190 (9.8%) | 259,828 (10%) | 267,395 (10%) | 272,683 (10%) | 283,709 (11%) | 298,076 (11%) | 308,210 (12%) | 316,159 (12%) | 322,623 (12%) | 329,053 (12%) | 331,173 (12%) |
| 80-89 | 137,356 (5.4%) | 138,453 (5.4%) | 138,755 (5.4%) | 140,703 (5.4%) | 142,363 (5.5%) | 144,319 (5.5%) | 146,879 (5.6%) | 149,531 (5.7%) | 151,850 (5.7%) | 154,379 (5.8%) | 155,454 (5.8%) | 159,120 (5.9%) |
| 90 and over | 33,263 (1.3%) | 34,540 (1.3%) | 35,423 (1.4%) | 35,946 (1.4%) | 36,707 (1.4%) | 36,970 (1.4%) | 36,750 (1.4%) | 37,118 (1.4%) | 37,210 (1.4%) | 37,986 (1.4%) | 37,556 (1.4%) | 37,155 (1.4%) |
| **BMI category** |  |  |  |  |  |  |  |  |  |  |  |  |
| Underweight | 15,287 (0.6%) | 15,526 (0.6%) | 16,239 (0.6%) | 16,712 (0.6%) | 16,552 (0.6%) | 16,724 (0.6%) | 16,494 (0.6%) | 17,241 (0.7%) | 17,215 (0.6%) | 12,147 (0.5%) | 13,860 (0.5%) | 15,697 (0.6%) |
| Normal weight | 194,598 (7.6%) | 195,786 (7.6%) | 198,018 (7.6%) | 195,291 (7.5%) | 188,343 (7.2%) | 188,362 (7.2%) | 184,571 (7.0%) | 184,329 (7.0%) | 185,076 (7.0%) | 124,922 (4.7%) | 144,108 (5.4%) | 164,356 (6.1%) |
| Obese | 250,480 (9.8%) | 250,273 (9.7%) | 248,689 (9.6%) | 245,806 (9.5%) | 247,580 (9.5%) | 257,747 (9.8%) | 262,665 (10.0%) | 265,649 (10%) | 273,755 (10%) | 193,053 (7.3%) | 228,082 (8.5%) | 272,582 (10%) |
| Overweight | 225,768 (8.8%) | 225,895 (8.8%) | 225,533 (8.7%) | 219,290 (8.4%) | 214,245 (8.2%) | 217,752 (8.3%) | 217,593 (8.3%) | 216,041 (8.2%) | 220,873 (8.3%) | 147,268 (5.6%) | 169,675 (6.3%) | 204,833 (7.6%) |
| Unknown | 1,875,824 (73%) | 1,889,141 (73%) | 1,901,575 (73%) | 1,919,374 (74%) | 1,940,054 (74%) | 1,939,323 (74%) | 1,950,105 (74%) | 1,956,315 (74%) | 1,952,266 (74%) | 2,168,773 (82%) | 2,121,617 (79%) | 2,026,594 (76%) |
| **Welsh Index of Multiple Deprivation 2019*** | | | | | | | | | | | | |
| 1. Most deprived | 487,251 (19%) | 490,215 (19%) | 492,264 (19%) | 493,963 (19%) | 496,692 (19%) | 500,346 (19%) | 503,565 (19%) | 505,138 (19%) | 507,807 (19%) | 507,777 (19%) | 511,864 (19%) | 515,640 (19%) |
| 2 | 503,126 (20%) | 505,724 (20%) | 506,696 (20%) | 506,996 (20%) | 508,415 (20%) | 510,505 (19%) | 512,241 (19%) | 513,971 (19%) | 516,096 (19%) | 515,394 (19%) | 520,491 (19%) | 521,636 (19%) |
| 3 | 536,153 (21%) | 539,440 (21%) | 542,268 (21%) | 542,110 (21%) | 544,714 (21%) | 546,898 (21%) | 548,324 (21%) | 550,228 (21%) | 552,804 (21%) | 550,919 (21%) | 559,283 (21%) | 559,652 (21%) |
| 4 | 519,339 (20%) | 522,407 (20%) | 525,871 (20%) | 528,193 (20%) | 530,410 (20%) | 533,545 (20%) | 536,580 (20%) | 538,699 (20%) | 540,181 (20%) | 540,586 (20%) | 547,621 (20%) | 548,127 (20%) |
| 5. Least deprived | 516,088 (20%) | 518,835 (20%) | 522,955 (20%) | 525,211 (20%) | 526,543 (20%) | 528,614 (20%) | 530,718 (20%) | 531,539 (20%) | 532,297 (20%) | 531,487 (20%) | 538,083 (20%) | 539,007 (20%) |
| **Rural urban classification** | | | | | | | | | | | | |
| Rural town and fringe | 333,545 (13%) | 335,146 (13%) | 336,279 (13%) | 337,315 (13%) | 338,004 (13%) | 339,508 (13%) | 341,208 (13%) | 342,240 (13%) | 343,101 (13%) | 343,425 (13%) | 347,199 (13%) | 348,363 (13%) |
| Rural town and fringe in a sparse setting | 102,466 (4.0%) | 102,958 (4.0%) | 102,944 (4.0%) | 102,656 (4.0%) | 102,757 (3.9%) | 102,871 (3.9%) | 103,016 (3.9%) | 103,476 (3.9%) | 103,484 (3.9%) | 102,826 (3.9%) | 104,221 (3.9%) | 103,893 (3.9%) |
| Rural village and dispersed | 174,970 (6.8%) | 175,467 (6.8%) | 176,431 (6.8%) | 176,540 (6.8%) | 177,102 (6.8%) | 177,844 (6.8%) | 178,925 (6.8%) | 179,594 (6.8%) | 180,110 (6.8%) | 180,136 (6.8%) | 183,109 (6.8%) | 182,843 (6.8%) |
| Rural village and dispersed in a sparse setting | 197,333 (7.7%) | 198,333 (7.7%) | 199,973 (7.7%) | 200,501 (7.7%) | 200,832 (7.7%) | 200,992 (7.7%) | 201,502 (7.7%) | 202,086 (7.7%) | 202,287 (7.6%) | 202,943 (7.7%) | 206,954 (7.7%) | 207,039 (7.7%) |
| Urban city and town | 1,701,029 (66%) | 1,711,555 (66%) | 1,721,514 (66%) | 1,726,898 (67%) | 1,736,067 (67%) | 1,746,810 (67%) | 1,755,215 (67%) | 1,760,630 (67%) | 1,769,016 (67%) | 1,766,365 (67%) | 1,785,148 (67%) | 1,791,254 (67%) |
| Urban city and town in a sparse setting | 52,614 (2.1%) | 53,162 (2.1%) | 52,913 (2.0%) | 52,563 (2.0%) | 52,012 (2.0%) | 51,883 (2.0%) | 51,562 (2.0%) | 51,549 (2.0%) | 51,187 (1.9%) | 50,468 (1.9%) | 50,711 (1.9%) | 50,670 (1.9%) |
| ^1^n (%) | | | | | | | | | | | | |

Supplementary Material Figure 1. Cohort construction.


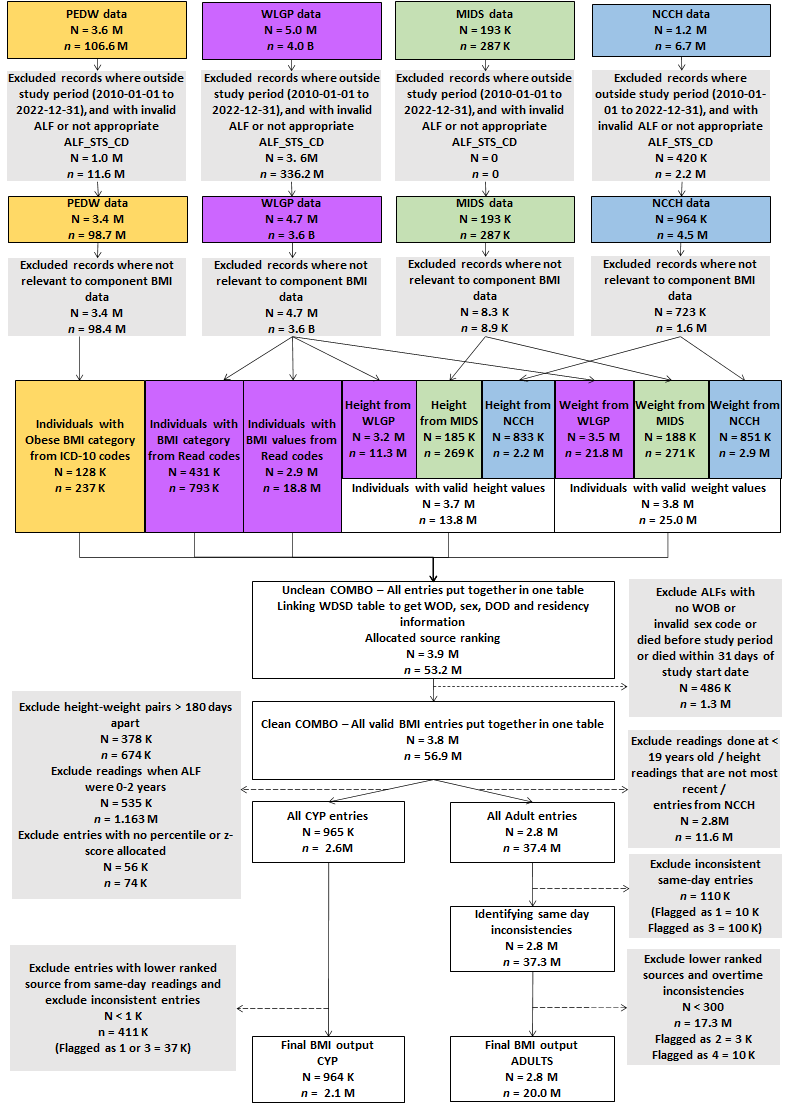


Supplementary Material Text 1.

**Same day readings**

For same-day entries with only BMI categories recorded, different BMI categories were flagged as “1”.

For same-day entries with BMI values present, these were arranged by the BMI value. Acceptable same-day variations of BMI values were set to 5%.

1. Entries with BMI values with more than a 5% difference and different BMI categories to the previous or next entry were flagged as “3”.
2. Entries with BMI values with *more* than 5% difference, but have the same BMI category, flag as “5”.
3. Entries with BMI values with *less* than 5% difference, but have different BMI categories, that is, they moved across the next category boundary, flag as “6”.

Flags 5 and 6 were used to identify entries within our consistency standards, i.e., same BMI categories and/or within 5% threshold. Adding these two flags allowed us to keep a record of the BMI category for the individual for that visit, which would otherwise removed as inconsistent in the first rule.

Example:

| Row | ALF | Date | BMI Category | Source rank | BMI value | Flag | Description |
| --- | --- | --- | --- | --- | --- | --- | --- |
| 1 | 1234 | 2019-01-01 | Underweight | 5 |  | 1 | Only BMI categories present and different categories recorded |
| 2 | 1234 | 2019-01-01 | Normal weight | 5 |  | 1 | Only BMI categories present and different categories recorded |
| 3 | 1234 | 2019-01-01 | Underweight | 1 | 18 | 3 | BMI values >5% different and different categories |
| 4 | 1234 | 2019-01-01 | Normal weight | 2 | 21 | 3 | BMI values >5% different and different categories |
| 5 | 1234 | 2019-01-01 | Normal weight | 1 | 21.5 |  | No flag |
| 6 | 1234 | 2019-01-01 | Normal weight | 2 | 24 | 5 | BMI values >5% different and same categories |
| 7 | 1234 | 2019-01-01 | Overweight | 2 | 25 | 6 | BMI values <5% different and different categories |

Note: In this fictional data example, we will remove rows 1-4 and keep rows 5-7 at this stage for the researchers to check later on. Going in to the next stage, only row 5 will be kept as this is the entry with the lowest source rank and lowest flag.

**Longitudinal readings**

If multiple acceptable entries were kept from the same-day cleaning, these will be arranged by source rank and flag. Only the entry with the lowest source rank and lowest flag will be kept for over-time cleaning.

Acceptable over-time variations of BMI category and values were set to .03%, equating to a 10% change within a 30-day period. BMI categories (numbered 1-4) with value change per day that was above the defined threshold were flagged as “2”. BMI values with more than 0.3% change in weight or BMI value per day were flagged as “4”.

These flags were not applied to the CYP cohort, allowing for developmental changes in this group.

Supplementary Material Figure 2 – trends of known BMI records over time for adult population over time.


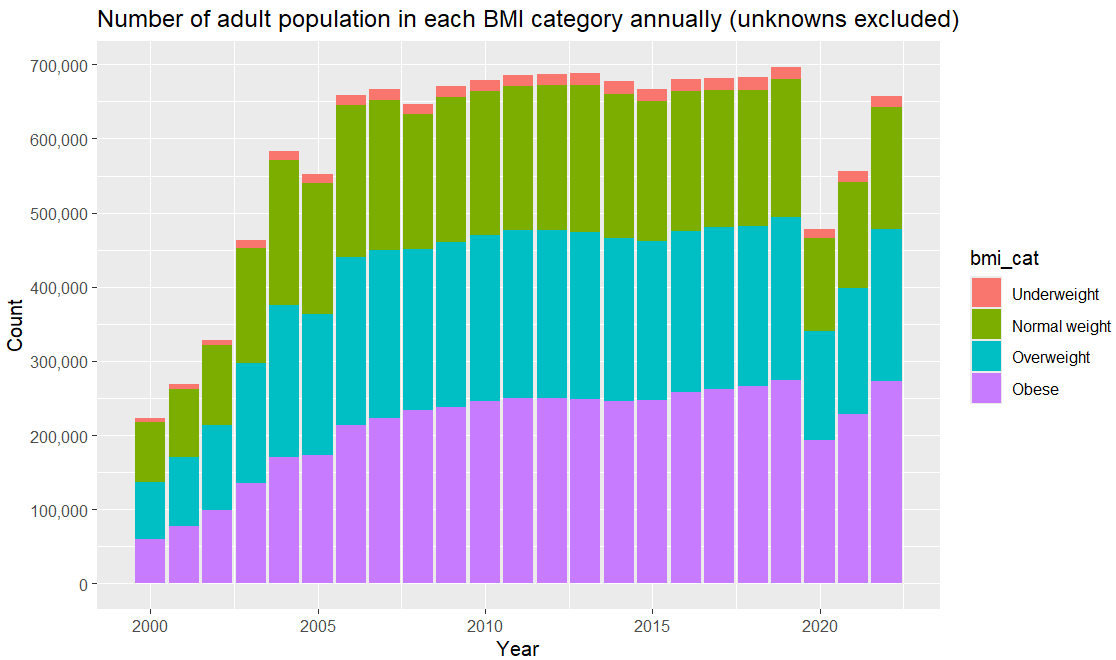

Supplement: online supplemental file 1 [file bmjopen-15-10-s001.docx]
