# Supplementary material for: Bridging the gap: development of a methodology for retrieving and harmonising body mass index (BMI) from population-level linked electronic health records
Source: BMJ Open. 2025 Oct 5;15(10):e103724. doi: 10.1136/bmjopen-2025-103724 (PMC12506101; doi:10.1136/bmjopen-2025-103724)
Supplement: Supplementary file 2 [file bmjopen-2025-103724.reviewer_comments.pdf]

PEER REVIEW HISTORY

BMJ Open publishes all reviews undertaken for accepted manuscripts. Reviewers are asked to complete a checklist review form and are provided with free text boxes to elaborate on their assessment. These free text comments are reproduced below.

ARTICLE DETAILS

Title (Provisional)

Bridging the gap: Development of a methodology for retrieving and harmonising Body Mass Index (BMI) from population-level linked electronic health records.

Authors

Childs, Michael Jeanne; Aldridge, Sarah J; Daniels, Helen; Davies, Gareth Ivor; Best, Victoria; Abbasizanjani, Hoda; Lyons, Ronan; Akbari, Ashley; Torabi, Fatemeh

---

VERSION 1 - REVIEW

**Reviewer**	**1**
**Name**	**Wood, Charles**
**Affiliation**	**Duke University, School of Medicine**
**Date**	**22-May-2025**
**COI**	**None**

This paper describes the attempt to combine BMI data from multiple different data sources and describe the methodology in detail while also comparing the prevalence of BMI data among children and adults across Wales and across different measurement settings in a 20+ year period. The authors do an excellent job of describing their process and decision-making around rules for inclusion and exclusion of valid data, which will allow an interested reader to replicate the processes, even if it is with another health system or population health data source. Their decision-making around this methodology is sound, albeit the paper lacks a deep enough discussion of the rationale for doing this, when the elephant in the room are the reasons for health systems or other sources of data collection not prioritizing BMI measurement routinely, as overall, only 10-20% of the population has a BMI recorded within a nearly 20 year period.

Specific comments:

Abstract conclusion – the first sentence seems to be less important than the second (methods). Although the primary aim and rationale of the analysis needs to be clarified (see below), the most apparently important conclusion is the use of this methodology for replication of the database combination.

Introduction:

Line 55: If BMI is collected selectively, as is detailed more in the discussion, then reporting categories of BMI across ages needs to be done with much more caution, or at least compared

among databases where the clear assumptions about the bias in measures towards healthier or sicker populations is clearer

“Multiple data sources” is used several times throughout the introduction, but should be described in detail earlier. Why use these particular data sources and how do they differ in terms of regularity of measurements, clinical context, etc.

Methods

Line 80: The section begins and the reader assumes that the rationale for this analysis was the development of the data harmonization methodologies. But a much clearer description of why this was needed and what the goals are for reporting this (i.e. what can an outside reader use from this for a similar problem) is needed. What is the main problem the authors are trying to address? It’s clear the main goal is not simply descriptive of the Wales population.

Line 150: 180 days is quite a large interval for children under 5, where BMI changes frequently, including decreases in raw BMI (nadir), so would suggest adjusting this methodology to a shorter default value.

Line 173: Please clarify what is “ordered based on ascending BMI values, ” “over-time readings,” and “lowest hierarchy of source rank.”

Line 191: The concept of “differing longitudinal preservation” needs clarification. What would be the reason for looking at why BMIs are preserved over time when there is little reliability that the current databases do anything resembling routine or universal BMI collection? Moreover, typically, the preservation of any BMI recorded at any point in the electronic health record is fairly automated, so I’m not clear why this needs to be ascertained other than as a way to indirectly understand clinician decision making about whether a weight and height were measured. I imagine most of the missingness for BMI in adults relates to heights not being immediately collected at regular intervals, but this can easily be solved by carrying forward the height, which of course cannot be done in children.

Results

Line 218: Clarify these percentages in parentheses. E.g. Is 8.4% the percentage of the Welsh population?

Line 229: For adults, it’s clear there is no real benefit in adding any data sources other than primary care, which is not surprising, per comment above on recorded height at other visits.

Discussion:

Line 264: The discussion suggests more frequent weight data is needed, but my guess is the height data is the missing factor. Why would weight not be recorded at every visit for every patient?

Line 286 – clarify why older BMIs might not be reliable. Also, “1.1 BMI value” is 1.1 kg/m^2? Children’s BMI changing is actually important to measure secular trends in BMI changes over time, which could be compared via sex- and age-specific percentiles (WHO or IOTF), so simply stating that not using older BMI values in children is a peculiar way to think about limitations,

unless it’s a similar issue to adult BMIs if there is a concern about data quality or measurement error.

**Reviewer**	**2**
**Name**	**Bann, David**
**Affiliation**	**University College London**
**Date**	**17-Jun-2025**
**COI**	**None**

This is a useful study/resource outlining BMI data derivation processes from electronic health records in Wales. The authors also provide the algorithms online to aid reproducibility and transparency; a very useful resource.

I have made some minor suggestions below. In general, making the article easier to follow for a non-familiar reader would be helpful throughout.

• Users may prefer, where possible, to access the continuous BMI values—since the categories lose information which can be undesirable in analyses. Please can you clarify.

• I found some of the manuscript hard to read due to the use of acronyms (e.g., can this be phrased in a higher level? “All four data sources: WLGP, PEDW, MIDS and NCCH” and spelt out in full in the Table 1?). At a high level please help the unfamiliar understand what the datasets are in the Methods/study design section—e.g., where they derive from.

• Please ensure that all figures are stand-alone understandable and clear to the non-familiar reader. e.g., figure 2 contains lots of acronyms; I was unable to follow figure 3.

• Re: removal of values judged to be extreme. Please specify what was defined as extreme. It is often preferable to flag rather than remove values to enable analysts to decide on which should be removed or retained.

• Table 2. I suggest separating out the unknown from the BMI category. So the %s in the BMI categories sum to 100.

• Discussion. “These findings underscore the need to optimise data integration strategies, particularly for enhancing BMI capture beyond primary care settings which is the main the source used in other studies”. Please rephrase to make clear what is needed and why for the unfamiliar reader.

• “This shows that only using primary care data may be detrimental for the CYP estimates but may be enough for the adults”. Please be more exacting. What do you mean by detrimental or ‘enough’? Again make clear for the unfamiliar reader.

• “We also note that the methodology applied same-day and over-time cleaning stages of cleaning for the adult cohort compared to only using same-day cleaning stage for the CYP cohort. This could account for the large difference in data from additional sources for the CYP cohort.” Is this something the authors could check.

• “data were consistently lower than the equivalent estimates” What are equivalent estimates?

• Figure 4 seems to show a very large change post pandemic. The change is so large that it seems to not be credible. Why is this found? (i.e., a methodological artifact and/or genuine change?).

• In supplementary material table 4, there appears to be a sizable fraction of unknown ethnicity at earlier years (20% in 2000 vs 5.7% in 2010). The authors may wish to note this. Particularly at younger ages the ethnicity may matter for BMI categorisation purposes given ethnic differences in height and body composition

---

VERSION 1 - AUTHOR RESPONSE

Reviewer: 1

Dr. Charles Wood, Duke University

Comments to the Author:

This paper describes the attempt to combine BMI data from multiple different data sources and describe the methodology in detail while also comparing the prevalence of BMI data among children and adults across Wales and across different measurement settings in a 20+ year period. The authors do an excellent job of describing their process and decision-making around rules for inclusion and exclusion of valid data, which will allow an interested reader to replicate the processes, even if it is with another health system or population health data source. Their decision-making around this methodology is sound, albeit the paper lacks a deep enough discussion of the rationale for doing this, when the elephant in the room are the reasons for health systems or other sources of data collection not prioritising BMI measurement routinely, as overall, only 10-20% of the population has a BMI recorded within a nearly 20 year period.

Response: Thank you for your thoughtful and encouraging feedback. We’re pleased that our effort, focused on the reproducibility aspect of this methodology, is clear, and you found our description of the methodology, including inclusion and exclusion criteria, and overall decision-making process clear and replicable. We also appreciate your recognition of the paper’s potential relevance to other health systems and population health data sources. We have also made the discussion point fuller; it now reads as follows:

> “While this work focuses on improving the capture and harmonisation of BMI data, it is important to acknowledge the broader systemic issue: BMI measurement is not routinely prioritised across all sectors of the health system. Despite its known associations with a wide range of chronic conditions and its role in public health surveillance, BMI remains under-recorded in routine clinical settings or at least in the coded data available. Only 10–20% of the population in Wales had a BMI recorded during the 20-year study period. This reflects a deeper structural challenge: BMI is often recorded opportunistically, typically during clinical encounters where weight-related issues are already suspected, rather than as part of standardised, proactive monitoring. Factors contributing to this include time constraints in primary care, limited incentive structures, lack of integration between different healthcare services, and an absence of clear national guidelines mandating regular BMI collection for all individuals. Moreover, healthcare professionals may be reluctant to raise weight-related topics without a clinical prompt, due to concerns about stigma or patient resistance. As such, our work is situated within this broader context of under-recording and seeks to address the methodological gap that results from these systemic practices. By demonstrating the added

> value of combining multiple data sources and preserving longitudinal BMI records, our aim is not only to improve research-quality data, but also to inform future policy and infrastructure changes that could enable more systematic and equitable BMI monitoring at the population level."

Specific comments:

Abstract conclusion – the first sentence seems to be less important than the second (methods). Although the primary aim and rationale of the analysis needs to be clarified (see below), the most apparently important conclusion is the use of this methodology for replication of the database combination.

Response: Thank you for your feedback. We have restructured this paragraph to highlight the importance of the method, which could be used across different trusted research environments for the reliable extraction of BMI data. It now reads as follows:

> "Conclusions - Our methodology provides a reproducible framework for extracting and harmonising BMI data from linked EHRs, adaptable across different trusted research environments. Our findings highlight substantial variations in BMI data availability and retention across CYP and adults, as well as time periods within EHRs in Wales. Wider adoption of this approach can enhance standardised approaches in using accessible measures, such as BMI, to assess disease risk in population-based studies, thereby strengthening public health initiatives and research efforts."

Introduction:

Line 55: If BMI is collected selectively, as is detailed more in the discussion, then reporting categories of BMI across ages needs to be done with much more caution, or at least compared among databases where the clear assumptions about the bias in measures towards healthier or sicker populations is clearer "Multiple data sources" is used several times throughout the introduction, but should be described in detail earlier. Why use these particular data sources and how do they differ in terms of regularity of measurements, clinical context, etc.

Response: Thank you for this important feedback. We agree that selective BMI recording introduces potential bias, and we have revised the introduction and discussion to emphasise this more clearly. We also revised the introduction to include mentions of "multiple data sources" and now provide earlier and more detailed descriptions of the data sources in the introduction, highlighting why they were chosen and how they differ in terms of measurement frequency, clinical context, and population coverage.

The introduction now reads as follows:

> "Moreover, BMI measurements in EHR are often recorded selectively, often during clinical encounters where weight is considered relevant, such as in cases involving comorbid conditions or those attending primary care for weight-related concerns (5,8). This results in BMI data being more complete for individuals with higher healthcare utilisation or specific clinical needs, introducing potential bias when using EHR-derived BMI data for population-level research (9–11). Consequently, reporting BMI categories across age groups should be approached with caution, as these data may overrepresent individuals who are older, sicker, or already identified as being at risk."

The discussion section reads as follows:

> “A key strength of our approach lies in the integration of multiple data sources, each differing in the frequency and context of BMI recording. This multi-source strategy helps mitigate some of the bias introduced by selective BMI measurement, particularly among individuals with greater healthcare utilisation. General practice data contributed the majority of adult BMI records, reflecting opportunistic recording during routine or condition-specific visits, while other data sources such as MIDS and NCCH were focused on specific prgrammes like maternity care and neonatal health, resulting in more structured and consistent BMI measurements across the population subgroups.”

Methods

Line 80: The section begins and the reader assumes that the rationale for this analysis was the development of the data harmonization methodologies. But a much clearer description of why this was needed and what the goals are for reporting this (i.e. what can an outside reader use from this for a similar problem) is needed. What is the main problem the authors are trying to address? It’s clear the main goal is not simply descriptive of the Wales population.

Response: Thank you for this helpful comment. We have clarified the rationale and goals of our study in the revised manuscript. Specifically, we added the following statements to our methodology section:

> "In using different data sources, we aim to increase the population coverage of BMI data for health research in Wales."

> "This approach supports researchers in either applying the algorithm to their respective cohorts or using the RRDA to extract harmonised BMI data, enhancing the reliability and comparability of BMI as a research variable."

We also acknowledge your point regarding the 180-day interval for young children. We have revised the methodology to allow users to set a shorter interval for children under 5, better reflecting the rapid changes in BMI during early childhood.

Line 150: 180 days is quite a large interval for children under 5, where BMI changes frequently, including decreases in raw BMI (nadir), so would suggest adjusting this methodology to a shorter default value.

Response: Thank you for this comment. We agree that BMI can change rapidly in early childhood; however, in the context of population-level data availability and routine practice in Wales, BMI is not measured frequently enough to justify a shorter interval. Routine measurements for children under five are typically spaced at intervals of more than 180 days, unless there is a clinical indication. For example, in Carmarthenshire, Health Visitors conduct developmental reviews—including height and weight—at approximately 15 months, 27 months, and 3.5 years of age. Additionally, the Child Measurement Programme (Wales) collects standardised measurements at school entry (ages 4–5) and again in Year 4 (ages 8–9), which are recorded in the National Community Child Health data (NCCH), used in this study.

Given these long intervals between measurements, our selection of a 180-day threshold to identify distinct BMI records is a conservative and context-appropriate choice. Using a shorter interval would not reflect routine clinical practice and may lead to unnecessary fragmentation of valid

longitudinal records. That said, we acknowledge that optimal thresholds may vary across settings, particularly in contexts with more frequent or structured measurements. The algorithm we developed is fully customisable, and users can modify the time interval based on the needs and characteristics of their specific population or data.

Line 173: Please clarify what is “ordered based on ascending BMI values, ” “over-time readings,” and “lowest hierarchy of source rank.”

Response: Thank you for bringing this point to our attention. We have clarified these terms in the revised manuscript as follows:

> "For individuals with multiple BMI category recordings on the same day, values were ordered by ascending BMI and BMI category—i.e., each measurement is listed in increasing order of BMI to help identify duplicates or inconsistencies."
>
> "We applied a two-stage cleaning process: (1) same-day readings, and (2) over-time readings, BMI measurements recorded on different dates, to address both duplicate and longitudinal inconsistencies."
>
> "In Stage 4, when multiple records from different sources existed, we retained the BMI value from the source with the highest reliability, based on the source hierarchy detailed in Table 1. A 'lower rank' refers to a more trusted or prioritised source in that hierarchy.”

Line 191: The concept of “differing longitudinal preservation” needs clarification. What would be the reason for looking at why BMIs are preserved over time when there is little reliability that the current databases do anything resembling routine or universal BMI collection? Moreover, typically, the preservation of any BMI recorded at any point in the electronic health record is fairly automated, so I’m not clear why this needs to be ascertained other than as a way to indirectly understand clinician decision making about whether a weight and height were measured. I imagine most of the missingness for BMI in adults relates to heights not being immediately collected at regular intervals, but this can easily be solved by carrying forward the height, which of course cannot be done in children.

Response: Thank you for this helpful comment. We have clarified the concept of "longitudinal preservation" in all sections of the revised manuscript. While BMI records are retained within electronic health records, they are not automatically carried forward for inclusion in data extracts based on fixed time windows. For example, if an individual's last recorded BMI was in 2014, it would not appear in the 2022 data unless the extraction period extends back far enough. This highlights the importance of clearly defining the preservation window for the use of longitudinal data. It now reads as follows:

> Abstract: “Preserving BMI information by retaining the most recently recorded BMI over one, five, and 23-year intervals from 2022 showed coverage rates of 9%, 25% and 64% respectively for CYP, and 25%, 31% and 74% respectively for adults.”
>
> “Finally, to assess how assumptions about data availability over time affect BMI coverage estimates, and investigate the effect of differing, we compared longitudinal preservation of BMI records on the cohort we looked at three distinct observation windows anchored to the follow-up periods. We investigated the BMI coverage for the end day of the study (31st December 2022): preserving only the most recent entry from the past in one year (start date

1st January 2022), the past five years (start date 1st January 2018) and the entire study period (start date 1st January 2000). This approach does not assume that BMI is routinely or universally recorded across the population but instead reflects how BMI data are often used in research."

Regarding height, we agree that it is often not routinely recorded in adults. Our methodology addresses this by carrying forward the most recent height record for adults and pairing it with available weight data from all sources. It's important to note that adults may lose height as they age, which can introduce error into the carry-forward method if this is implemented on long time intervals(Ji et al., 2023). We acknowledge that this approach is not appropriate for children, as their height and BMI change rapidly and require time-specific measurements. We have now added this in our discussion section reading as follow:

Ji, S., Lee, E., Kim, B. J., Baek, J. Y., Yi, Y., Jang, I. Y., & Jung, H. W. (2023). Height loss as an indicator of ageing through its association with frailty and sarcopenia: An observational cohort study. *Archives of Gerontology and Geriatrics*, *110*, 104916. https://doi.org/10.1016/J.ARCHGER.2022.104916

Results

Line 218: Clarify these percentages in parentheses. E.g. Is 8.4% the percentage of the Welsh population?

Response: Thank you for this point. We have added the clarification, and this sentence now reads:

"Using phenotyped BMI categories, as recorded in patients' primary care data using Read codes, we identified 431,156 unique individuals (8.4% of the total population) between 2000 and 2022."

Line 229: For adults, it's clear there is no real benefit in adding any data sources other than primary care, which is not surprising, per comment above on recorded height at other visits.

Response: Thank you for this observation. We agree that primary care is the dominant source of BMI information for adults, as reflected in our findings. This aligns with expectations, given that height and weight are more routinely captured during primary care consultations than in hospital or maternity settings. However, our inclusion of additional sources, though contributing <2% of unique adult records, was important to confirm this distribution empirically and ensure that no meaningful volume of BMI data from non-primary care settings was overlooked. Notably, using phenotyped BMI categories from Read codes in primary care alone, we identified 431,156 unique adults (8.4% of the Welsh population) over the 22-year study period, which underscores both the utility and limitations of relying solely on this source. While the benefit of adding other sources may be minimal for adults at present, this could become more valuable in future if natural language processing (NLP) techniques allow extraction of height and weight information from clinical correspondence, potentially offering more recent or contextually relevant data, we maintain that a comprehensive, inclusive approach is essential when designing harmonisation methodologies intended for reuse across age groups and use cases.

Discussion:

Line 264: The discussion suggests more frequent weight data is needed, but my guess is the height data is the missing factor. Why would weight not be recorded at every visit for every patient?

Response: Thank you for this comment. From the data we have extracted, there were more unique individuals, as well as more records with weight measurements, compared to those with height measurements (Figure 2). Height as a trait is also more stable than weight, particularly in the adult population; therefore we were able to use the most recent height recording and match it to the available weight records. Previous studies have also demonstrated that weight is not routinely recorded in the UK, and that it is more likely to be recorded in the older population and those with clinical needs (Nicholson et al., 2019; McLauhglin et al., 2017).

Line 286 – **clarify why older BMIs might not be reliable**. Also, “1.1 BMI value” is 1.1 kg/m^2? Children’s BMI changing is actually important to measure secular trends in BMI changes over time, which could be compared via sex- and age-specific percentiles (WHO or IOTF), so simply stating that not using older BMI values in children is a peculiar way to think about limitations, unless it’s a similar issue to adult BMIs if there is a concern about data quality or measurement error.

Response: Thank you for this helpful comment. We also corrected the phrasing to read "1.1 kg/m^2" instead of "1.1 BMI value" for clarity, and also added a fuller statement about the use of older BMI records, which reads as follows:

> “ Bishay et. al., 2021 (30) observed that older BMI records for individuals, (3< years), tend to be on average 1.1 kg/m2 lower than more recent measurements, highlighting the potential for underestimation of BMI value when relying on historic records. While the study did not conclude that older BMI data are inherently unreliable, this finding underscores the need for caution when interpreting longitudinal BMI trends, particularly in adult populations.”

Reviewer: 2

Dr. David Bann, University College London

Comments to the Author:

This is a useful study/resource outlining BMI data derivation processes from electronic health records in Wales. The authors also provide the algorithms online to aid reproducibility and transparency; a very useful resource.

I have made some minor suggestions below. In general, making the article easier to follow for a non-familiar reader would be helpful throughout.

Response: Thank you for your positive and supportive feedback. We’re pleased to hear that you found the study and the openly shared algorithms useful. We appreciate your suggestions and have made revisions to improve clarity and accessibility for readers who may be less familiar with the topic.

• Users may prefer, where possible, to access the continuous BMI values—since the categories lose information which can be undesirable in analyses. Please can you clarify.

Response: Thank you for this comment. The final table generated by the methodology contains all the BMI information extracted for each individual, including height, weight, BMI value (recorded and calculated), and BMI categories (recorded and calculated) for each person, each day they had their BMI data recorded. Users could therefore access the continuous BMI values that were extracted/calculated by the methodology.

• I found some of the manuscript hard to read due to the use of acronyms (e.g., can this be phrased in a higher level? "All four data sources: WLGP, PEDW, MIDS and NCCH" and spelt out in full in the Table 1?). At a high level please help the unfamiliar understand what the datasets are in the Methods/study design section—e.g., where they derive from.

Response: Thank you for this point. We appreciate that the manuscript uses a fair amount of acronyms due to the nature of the data sources involved. We have spelt out the names in full in the Study Design section and have used acronyms henceforth. For Table 1, and all of our figures we have now included a footnote spelling out the acronyms for further clarity. Additionally, we have included more high level details regarding the data sources in the methods section reading as below:

> "This is an observational study where we developed a methodology that extracted BMI components from four data sources including height, weight, BMI values and categories from primary care records in Welsh Longitudinal General Practice (WLGP) data (16), diagnoses of obesity from secondary care (hospital admission) records in Patient Episode Database for Wales (PEDW) (17), height and weight during pregnancy in Maternity Indicators Dataset (MIDS) (18), and height and weight from the Child Measurement Programme (Wales) in National Community Child Health (NCCH) data."

• Please ensure that all figures are stand-alone understandable and clear to the non-familiar reader. e.g., figure 2 contains lots of acronyms; I was unable to follow figure 3.

Response: Thank you for this comment. We have now revised all our figures and added sufficient information about the data used in the methodology to make them understandable as standalone elements.

• Re: removal of values judged to be extreme. Please specify what was defined as extreme. It is often preferable to flag rather than remove values to enable analysts to decide on which should be removed or retained.

Response: Thank you for this point. The method generates a table with all the data extracted from each stage. In particular, for Stage 1, data tables are generated to extract BMI values, categories, and height and weight. These are then collated into a long table after extreme values have been removed. The user, if they wish, could set the method to keep these tables for later inspection. For the purposes of this paper, the default tables provided are the table with the flagged inconsistent data and the final cleaned table.

Extreme values - We have clarified the BMI values used in this study. The height and weight calculations are provided in Supplementary Table 1.

• Table 2. I suggest separating out the unknown from the BMI category. So the %s in the BMI categories sum to 100.

Response: Thank you for your feedback. However, on balance, we think that keeping the unknown BMIs in the tables adds to population level insights and highlights the importance of our method, as we aimed to reduce the percentage of unknown BMIs using our methodology.

• Discussion. "These findings underscore the need to optimise data integration strategies, particularly for enhancing BMI capture beyond primary care settings which is the main the source used in other studies". Please rephrase to make clear what is needed and why for the unfamiliar reader.

Response: Thank you for this point. We have added a statement to discussion, clarify this which reads as:

> "In other words, recording of BMI related data in settings outside the primary care, particularly in adults, could be improved to provide a more significant supplement to increase BMI coverage"

• "This shows that only using primary care data may be detrimental for the CYP estimates but may be enough for the adults". Please be more exacting. What do you mean by detrimental or 'enough'? Again make clear for the unfamiliar reader.

Response: Thank you for this comment. We have added the following statements to clarify:

> "This shows that unique individuals from maternity records and hospital admissions only accounted for less than 2% of both cohorts. As the adult population did not have another data source dedicated to collecting BMI data, using data from primary care would be sufficient for BMI extraction. However, the community children's data source (NCCH) provided a significant proportion of children's BMI data supplementing the primary care source, therefore only utilising primary care data would not be representative of the CYP population"

• "We also note that the methodology applied same-day and over-time cleaning stages of cleaning for the adult cohort compared to only using same-day cleaning stage for the CYP cohort. This could account for the large difference in data from additional sources for the CYP cohort." Is this something the authors could check.

Response: Thank you for the suggestion. We have reviewed the cleaning stages applied to both cohorts and confirmed that the difference in methodology likely contributes to the variation in data from additional sources for the CYP cohort. We have added this clarification to the manuscript.

• "data were consistently lower than the equivalent estimates" What are equivalent estimates?

Response: Thank you, we have clarified this: equivalent estimates in terms of the previous studies . The same note applies to the Bishay et al. (2019) reference.

• Figure 4 seems to show a very large change post pandemic. The change is so large that it seems to not be credible. Why is this found? (i.e., a methodological artifact and/or genuine change?).

Response: We agree that this is a large change, to explain this change and ensure that it is not an artifact of method we conducted some investigations. Looking at overall number of records in the dataset and observed the same trend as in figure 4. This drop is likely due to the the changes to GP contacts and weight measurements during COVID-19 pandemic, and its impact on healthcare

utilisation and interaction with services from individuals throughout and following the COVID-19 pandemic, as well as the associated measures and lockdowns in the UK. We now include the following figure in supplementary material to inform readers who might have the same concern and be interested in impelementing our methodology in another dataset.

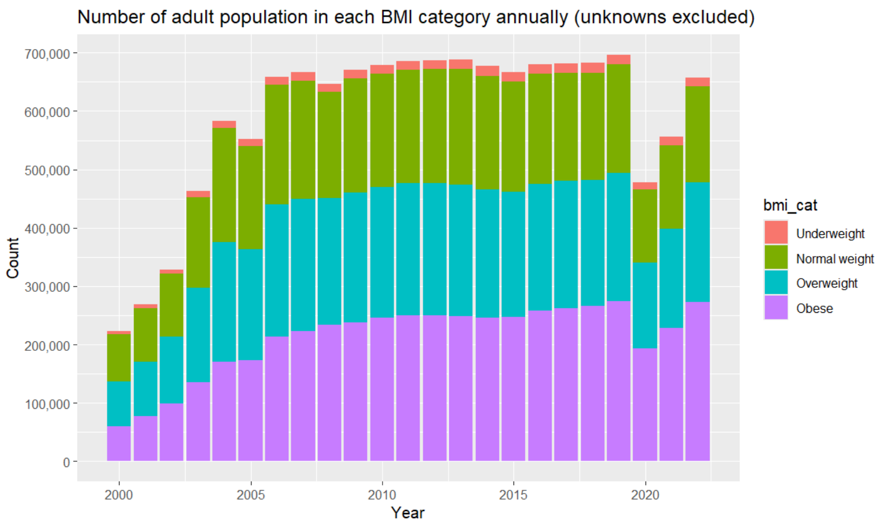

Supplementary material figure 2 – trends of known BMI records over time for adult population over time.

• In supplementary material table 4, there appears to be a sizable fraction of unknown ethnicity at earlier years (20% in 2000 vs 5.7% in 2010). The authors may wish to note this. Particularly at younger ages the ethnicity may matter for BMI categorisation purposes given ethnic differences in height and body composition

Response: Thank you for this important observation. We agree that the high proportion of unknown ethnic groups in earlier years, especially around 20% in 2000 compared to 5.7% in 2010, is noteworthy. Given its relevance to BMI categorisation due to ethnic group differences in growth and body composition, we have now brought Supplementary Table 4 into the main manuscript and included a discussion highlighting this limitation and its potential impact on our findings. It reads as follow:

> “Only 10–20% of the population in Wales had a BMI recorded during the 20-year study period. This reflects a deeper structural challenge: BMI is often recorded opportunistically, typically during clinical encounters where weight-related issues are already suspected, rather than as part of standardised, proactive monitoring. Factors contributing to this include time constraints in primary care, limited incentive structures, lack of integration between different healthcare services, and an absence of clear national guidelines mandating regular BMI collection for all individuals. (See supplementary Material Figure 2) ”

Reviewer: 1

Competing interests: not applicable

Reviewer: 2

Competing interests: N/A

VERSION 2 - REVIEW

**Reviewer**	**1**
**Name**	**Wood, Charles**
**Affiliation**	**Duke University, School of Medicine**
**Date**	**22-Aug-2025**
**COI**	

I'd like to thank the authors for their diligent addressing of my questions and comments. The responses are thorough and thoughtful and additions have improved the paper. I have just a few remaining comments, which are minor:

Please define CYP at the first appearance in the main manuscript. I realize it is defined in the abstract, but should be defined again in the full manuscript at first appearance.

Many of us believe that the use of BMI under 2 is scientifically valid, and so would suggest referring to use of BMI under 2 as “not routine,” instead of “not recommended.” It is true the US CDC does not suggest routine monitoring of BMI under 2, but the WHO does provide references for use and it can indeed be used.

I am still not totally sure about the reason for ordering potentially duplicate BMIs in ascending order. How does this help identify duplicates or inconsistencies? I would be just as concerned about the validity of duplicate BMIs from multiple data sources or the same data source on one specific day regardless of how they are ordered. Could duplicate BMIs be simply averaged? Or flagged without ordering in some way? In other words, the “true” BMI might be higher or lower, and depends on relative distance from the previous BMIs and subsequent BMIs more than discrepancies in category or magnitude of differences. You could refer to the methodological details in Carrie Daymont’s automated cleaning algorithm growthcleanr for details on how these often erroneous repeated measures are dealt with using moving averages, which could be a good solution: https://github.com/carriedaymont/growthcleanr

VERSION 2 - AUTHOR RESPONSE

Reviewer: 1

Dr. Charles Wood, Duke University

Comments to the Author:

I'd like to thank the authors for their diligent addressing of my questions and comments. The responses are thorough and thoughtful and additions have improved the paper.

Response: We thank Dr. Wood for their generous feedback and for recognising our efforts to address the comments in the previous round. We greatly appreciate the time and expertise devoted to reviewing our work, and we are pleased that the revisions have improved the presentation of our work for the readership of BMJ Open.

I have just a few remaining comments, which are minor:

Please define CYP at the first appearance in the main manuscript. I realize it is defined in the abstract, but should be defined again in the full manuscript at first appearance.

Response: Thank you for this helpful observation, we have now added the definition of CYP (children and young people) at its first appearance in the main manuscript in page 7 line 2, in addition to the abstract, to ensure clarity for readers.

Many of us believe that the use of BMI under 2 is scientifically valid, and so would suggest referring to use of BMI under 2 as "not routine," instead of "not recommended." It is true the US CDC does not suggest routine monitoring of BMI under 2, but the WHO does provide references for use and it can indeed be used.

Response: Thank you for this important clarification. We have revised the manuscript to describe the use of BMI under 2 years as "not routine", rather than "not recommended" in line 9 of page 10, to better reflect both CDC and WHO perspectives. In addition, we have added a note in the Discussion on page 18 line 17-21 acknowledging that although BMI for age is not routinely used under 2 years in the US (CDC guidance), the WHO provides growth standards that allow its use in the age group. We have cited relevant references to WHO[1] and CDC[2] recommendations that support these points:

1. de Onis, M., Adelheid, O., Borghi, E., Siyam, A. & Pinol, A. World Health Organization Growth Standards. *WHO* 5–6 (2006).
2. Using WHO Growth Standard Charts | Growth Chart Training | CDC. https://www.cdc.gov/growth-chart-training/hcp/using-growth-charts/who-using.html?utm_source=chatgpt.com.

I am still not totally sure about the reason for ordering potentially duplicate BMIs in ascending order. How does this help identify duplicates or inconsistencies? I would be just as concerned about the validity of duplicate BMIs from multiple data sources or the same data source on one specific day regardless of how they are ordered. Could duplicate BMIs be simply averaged? Or flagged without ordering in some way? In other words, the "true" BMI might be higher or lower, and depends on relative distance from the previous BMIs and subsequent BMIs more than discrepancies in category or magnitude of differences. You could refer to the methodological details in Carrie Daymont's automated cleaning algorithm growthcleanr for details on how these often erroneous repeated measures are dealt with using moving averages, which could be a good solution: https://github.com/carriedaymont/growthcleanr

Response: Thank you for this precise feedback. We deal with duplicated records after collating all measurements from all available data sources into a single master table;

therefore, while ranking sources is highly relevant in the context of the data sources we used in this paper, we have made it clear in the manuscript that users could choose to apply the same ranking across all sources if this better suits their analytic context.

Our approach followed a two-stage process:

Same-day duplicates: For individuals with multiple BMI values recorded on the same day, ordering values in ascending order allowed us to systematically identify extreme or implausible entries (e.g., obvious data entry errors) and compare them against more plausible same-day measures. This was intended as a pragmatic consistency check, not as a method of inferring the "true" BMI.

Longitudinal consistency: We then reviewed BMI records over time to identify implausible fluctuations.

In cases where multiple same-day records originated from different healthcare data sources, we retained the BMI from the most reliable source according to our predefined hierarchy (Table 1). This reflects the reality of healthcare data capture in Wales, where BMI is recorded across diverse settings (e.g., primary care, secondary care, child health systems). However, recognising the reviewer's point, we have clarified that users of our approach could elect to apply equal weighting across sources rather than using a source hierarchy.

We also agree with the reviewer that alternative approaches, such as averaging, flagging, or applying automated algorithms (e.g., growthcleanr, Daymont et al.), represent valuable methods. To acknowledge this, we have covered this fully in page 19 lines 14-20 in the Discussion highlighting that methods such as growthcleanr[3], which use moving averages to address repeated or erroneous measures, may provide a robust solution in certain contexts.

3. Daymont, C. *et al.* Automated identification of implausible values in growth data from pediatric electronic health records. *J. Am. Med. Informatics Assoc.* **24**, 1080–1087 (2017).

We thank you once again for the opportunity to revise and resubmit our work. We very much appreciate the time and effort invested by the reviewer and the editorial team, and we hope that our responses and revisions demonstrate our commitment to addressing all points thoroughly.
